# An extended U2AF^65^–RNA-binding domain recognizes the 3′ splice site signal

**DOI:** 10.1038/ncomms10950

**Published:** 2016-03-08

**Authors:** Anant A. Agrawal, Enea Salsi, Rakesh Chatrikhi, Steven Henderson, Jermaine L. Jenkins, Michael R. Green, Dmitri N. Ermolenko, Clara L. Kielkopf

**Affiliations:** 1 Center for RNA Biology and Department of Biochemistry and Biophysics, University of Rochester School of Medicine and Dentistry, Rochester, New York 14642, USA; 2 Howard Hughes Medical Institute and Programs in Gene Function and Expression and Molecular Medicine, University of Massachusetts Medical School, Worcester, Massachusetts 01655, USA

## Abstract

How the essential pre-mRNA splicing factor U2AF^65^ recognizes the polypyrimidine (Py) signals of the major class of 3′ splice sites in human gene transcripts remains incompletely understood. We determined four structures of an extended U2AF^65^–RNA-binding domain bound to Py-tract oligonucleotides at resolutions between 2.0 and 1.5 Å. These structures together with RNA binding and splicing assays reveal unforeseen roles for U2AF^65^ inter-domain residues in recognizing a contiguous, nine-nucleotide Py tract. The U2AF^65^ linker residues between the dual RNA recognition motifs (RRMs) recognize the central nucleotide, whereas the N- and C-terminal RRM extensions recognize the 3′ terminus and third nucleotide. Single-molecule FRET experiments suggest that conformational selection and induced fit of the U2AF^65^ RRMs are complementary mechanisms for Py-tract association. Altogether, these results advance the mechanistic understanding of molecular recognition for a major class of splice site signals.

## Abstract (web summary)

The pre-mRNA splicing factor U2AF65 recognizes 3′ splice sites in human gene transcripts, but the details are not fully understood. Here, the authors report U2AF65 structures and single molecule FRET that reveal mechanistic insights into splice site recognition.

## Graphical abstract

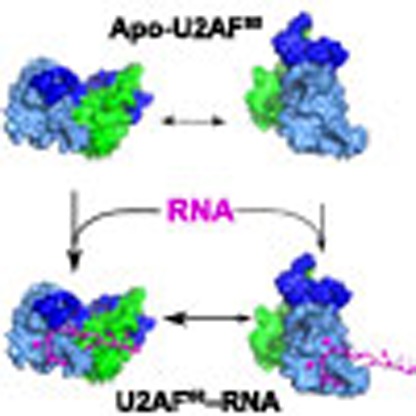

The differential skipping or inclusion of alternatively spliced pre-mRNA regions is a major source of diversity for nearly all human gene transcripts^1^. The splice sites are marked by relatively short consensus sequences and are regulated by additional pre-mRNA motifs (reviewed in ref. 2). At the 3′ splice site of the major intron class, these include a polypyrimidine (Py) tract comprising primarily Us or Cs, which is preceded by a branch point sequence (BPS) that ultimately serves as the nucleophile in the splicing reaction and an AG-dinucleotide at the 3′ splice site junction. Disease-causing mutations often compromise pre-mRNA splicing (reviewed in refs 3, 4), yet *a priori* predictions of splice sites and the consequences of their mutations are challenged by the brevity and degeneracy of known splice site sequences. High-resolution structures of intact splicing factor–RNA complexes would offer key insights regarding the juxtaposition of the distinct splice site consensus sequences and their relationship to disease-causing point mutations.

The early-stage pre-mRNA splicing factor U2AF^65^ is essential for viability in vertebrates and other model organisms (for example, ref. 5). A tightly controlled assembly among U2AF^65^, the pre-mRNA, and partner proteins sequentially identifies the 3′ splice site and promotes association of the spliceosome, which ultimately accomplishes the task of splicing^2^. Initially U2AF^65^ recognizes the Py-tract splice site signal^6^. In turn, the ternary complex of U2AF^65^ with SF1 and U2AF^35^ identifies the surrounding BPS^7^,^8^ and 3′ splice site junctions^9^,^10^,^11^. Subsequently U2AF^65^ recruits the U2 small nuclear ribonucleoprotein particle (snRNP) and ultimately dissociates from the active spliceosome.

Biochemical characterizations of U2AF^65^ demonstrated that tandem RNA recognition motifs (RRM1 and RRM2) recognize the Py tract^12^,^13^ (Fig. 1a). Milestone crystal structures of the core U2AF^65^ RRM1 and RRM2 connected by a shortened inter-RRM linker (dU2AF^65^1,2) detailed a subset of nucleotide interactions with the individual U2AF^65^ RRMs^14^,^15^. A subsequent NMR structure^16^ characterized the side-by-side arrangement of the minimal U2AF^65^ RRM1 and RRM2 connected by a linker of natural length (U2AF^65^1,2), yet depended on the dU2AF^65^1,2 crystal structures for RNA interactions and an *ab initio* model for the inter-RRM linker conformation. As such, the molecular mechanisms for Py-tract recognition by the intact U2AF^65^–RNA-binding domain remained unknown. Here, we use X-ray crystallography and biochemical studies to reveal new roles in Py-tract recognition for the inter-RRM linker and key residues surrounding the core U2AF^65^ RRMs. We use single-molecule Förster resonance energy transfer (smFRET) to characterize the conformational dynamics of this extended U2AF^65^–RNA-binding domain during Py-tract recognition.

## Results

### Cognate U2AF^65^–Py-tract recognition requires RRM extensions

**Figure 1:**
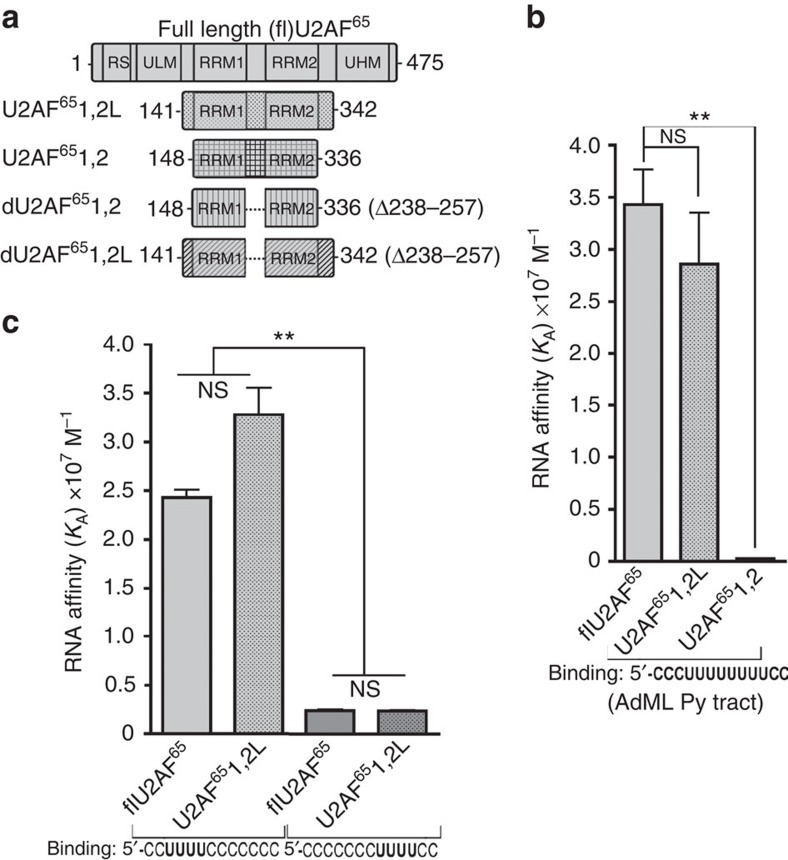
The intact U2AF^65^ RRM1/RRM2-containing domain and flanking residues are required for binding contiguous Py tracts. (**a**) Domain organization of full-length (fl) U2AF^65^ and constructs used for RNA binding and structural experiments. The N- and C-terminal residue numbers are indicated. An internal deletion (d, Δ) of residues 238–257 removes a portion of the inter-RRM linker from the dU2AF^65^1,2 and dU2AF^65^1,2L constructs. (**b**) Comparison of the apparent equilibrium affinities of various U2AF^65^ constructs for binding the prototypical AdML Py tract (5′-CCCUUUUUUUUCC-3′). The flU2AF^65^ protein includes a heterodimerization domain of the U2AF^35^ subunit to promote solubility and folding. The apparent equilibrium dissociation constants (*K*_D_) for binding the AdML 13mer are as follows: flU2AF^65^, 30±3 nM; U2AF^65^1,2L, 35±6 nM; U2AF^65^1,2, 3,600±300 nM. (**c**) Comparison of the RNA sequence specificities of flU2AF^65^ and U2AF^65^1,2L constructs binding C-rich Py tracts with 4U's embedded in either the 5′- (light grey fill) or 3′- (dark grey fill) regions. The *K*_D_'s for binding 5′-CCUUUUCCCCCCC-3′ are: flU2AF^65^, 41±2 nM; U2AF^65^1,2L, 31±3 nM. The *K*_D_'s for binding 5′-CCCCCCCUUUUCC-3′ are: flU2AF^65^, 414±12 nM; U2AF^65^1,2L, 417±10 nM. Bar graphs are hatched to match the constructs shown in **a**. The average apparent equilibrium affinity (*K*_A_) and s.e.m. for three independent titrations are plotted. The *P* values from two-tailed unpaired *t*-tests with Welch's correction are indicated as follows: ^**^P<0.01; NS, not significant, *P*>0.05. The purified protein and average fitted fluorescence anisotropy RNA-binding curves are shown in Supplementary Fig. 1. RRM, RNA recognition motif; RS, arginine-serine rich; UHM, U2AF homology motif; ULM, U2AF ligand motif.

The RNA affinity of the minimal U2AF^65^1,2 domain comprising the core RRM1–RRM2 folds (U2AF^65^1,2, residues 148–336) is relatively weak compared with full-length U2AF^65^ (Fig. 1a,b; Supplementary Fig. 1). Historically, this difference was attributed to the U2AF^65^ arginine–serine rich domain, which contacts pre-mRNA–U2 snRNA duplexes outside of the Py tract^17^,^18^,^19^. We noticed that the RNA-binding affinity of the U2AF^65^1,2 domain was greatly enhanced by the addition of seven and six residues at the respective N and C termini of the minimal RRM1 and RRM2 (U2AF^65^1,2L, residues 141–342; Fig. 1a). In a fluorescence anisotropy assay for binding a representative Py tract derived from the well-characterized splice site of the adenovirus major late promoter (AdML), the RNA affinity of U2AF^65^1,2L increased by 100-fold relative to U2AF^65^1,2 to comparable levels as full-length U2AF^65^ (Fig. 1b; Supplementary Fig. 1a–d). Likewise, both U2AF^65^1,2L and full-length U2AF^65^ showed similar sequence specificity for U-rich stretches in the 5′-region of the Py tract and promiscuity for C-rich regions in the 3′-region (Fig. 1c, Supplementary Fig. 1e–h).

### U2AF^65^-bound Py tract comprises nine contiguous nucleotides

**Figure 2:**
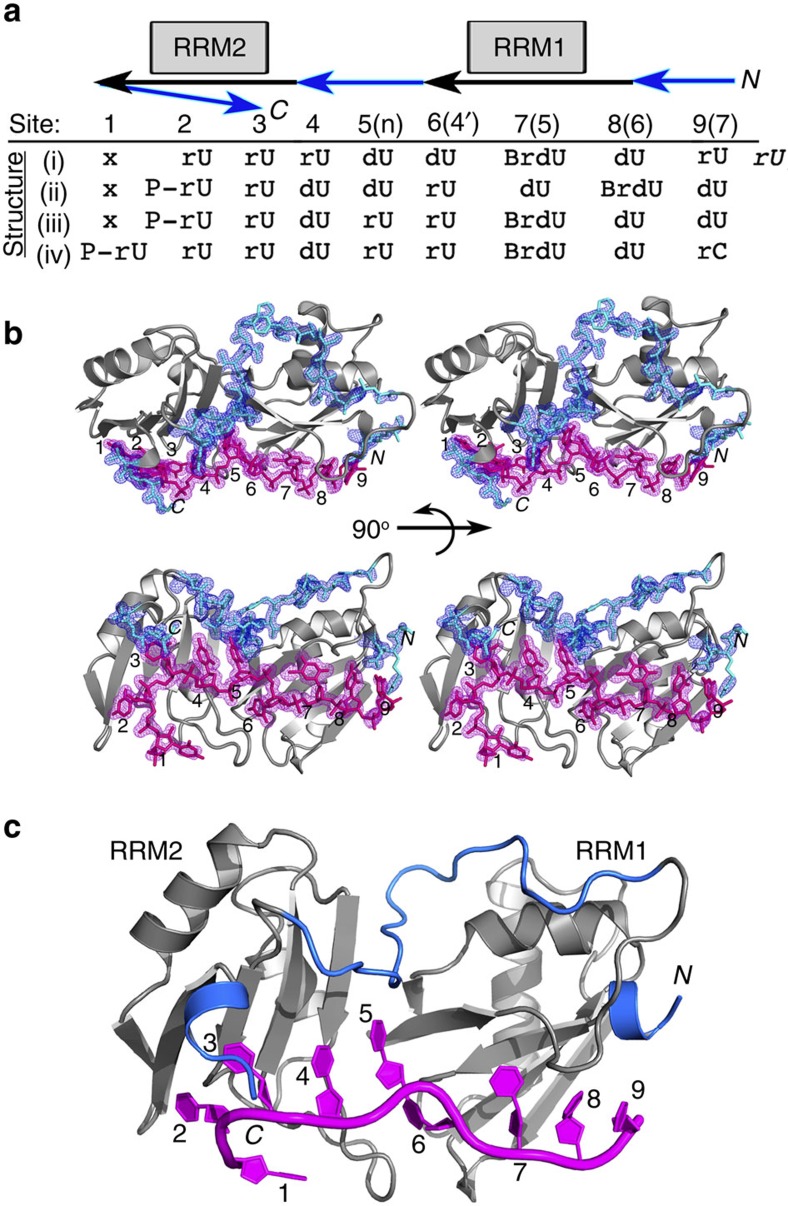
Structures of U2AF^65^1,2L recognizing a contiguous Py tract. (**a**) Alignment of oligonucleotide sequences that were co-crystallized in the indicated U2AF^65^1,2L structures. The regions of RRM1, RRM2 and linker contacts are indicated above by respective black and blue arrows from N- to C-terminus. For clarity, we consistently number the U2AF^65^1,2L nucleotide-binding sites from one to nine, although in some cases the co-crystallized oligonucleotide comprises eight nucleotides and as such leaves the first binding site empty. The prior dU2AF^65^1,2 nucleotide-binding sites are given in parentheses (site 4' interacts with dU2AF^65^ RRM1 and RRM2 by crystallographic symmetry). Italics, disordered in the structure. (**b**) Stereo views of a ‘kicked' 2|*F*_o_|−|*F*_c_| electron density map contoured at 1σ for the inter-RRM linker, N- and C-terminal residues (blue) or bound oligonucleotide of a representative U2AF^65^1,2L structure (structure *iv,* bound to 5′-(P)rUrUrUdUrUrU(BrdU)dUrC) (magenta). (**c**) Cartoon diagram of this structure. Crystallographic statistics are given in Table 1 and the overall conformations of U2AF^65^1,2L and prior dU2AF^65^1,2/U2AF^65^1,2 structures are compared in Supplementary Fig. 2. BrdU, 5-bromo-deoxy-uridine; d, deoxy-ribose; P-, 5′-phosphorylation; r, ribose.

To investigate the structural basis for cognate U2AF^65^ recognition of a contiguous Py tract, we determined four crystal structures of U2AF^65^1,2L bound to Py-tract oligonucleotides (Fig. 2a; Table 1). By sequential boot strapping (Methods), we optimized the oligonucleotide length, the position of a Br-dU, and the identity of the terminal nucleotide (rU, dU and rC) to achieve full views of U2AF^65^1,2L bound to contiguous Py tracts at up to 1.5 Å resolution. The protein and oligonucleotide conformations are nearly identical among the four new U2AF^65^1,2L structures (Supplementary Fig. 2a). The U2AF^65^1,2L RRM1 and RRM2 associate with the Py tract in a parallel, side-by-side arrangement (shown for representative structure iv in Fig. 2b,c; Supplementary Movie 1). An extended conformation of the U2AF^65^ inter-RRM linker traverses across the α-helical surface of RRM1 and the central β-strands of RRM2 and is well defined in the electron density (Fig. 2b). The extensions at the N terminus of RRM1 and C terminus of RRM2 adopt well-ordered α-helices. Both RRM1/RRM2 extensions and the inter-RRM linker of U2AF^65^1,2L directly recognize the bound oligonucleotide. We compare the global conformation of the U2AF^65^1,2L structures with the prior dU2AF^65^1,2 crystal structure^15^ and U2AF^65^1,2 NMR structure^16^ in the Supplementary Discussion and Supplementary Fig. 2.

**Figure 3:**
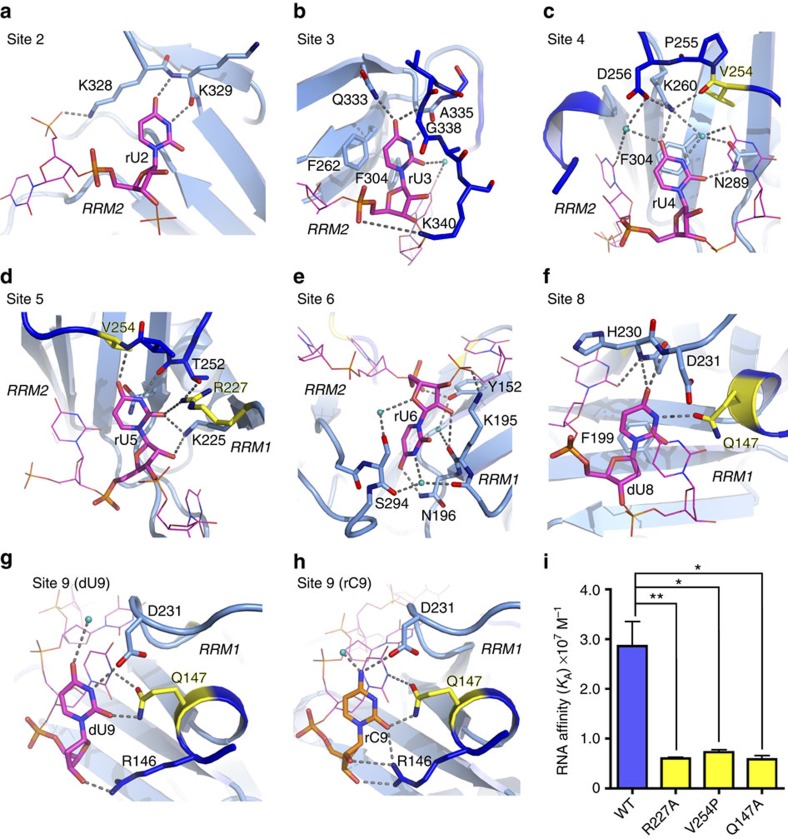
Representative views of the U2AF^65^1,2L interactions with each new nucleotide of the bound Py tract. New residues of the U2AF^65^1,2L structures are coloured a darker shade of blue, apart from residues that were tested by site-directed mutagenesis, which are coloured yellow. The nucleotide-binding sites of the U2AF^65^1,2L and prior dU2AF^65^1,2 structure are compared in Supplementary Fig. 3a–h. The first and seventh U2AF^65^1,2L-binding sites are unchanged from the prior dU2AF^65^1,2–RNA structure and are portrayed in Supplementary Fig. 3a,f. The four U2AF^65^1,2L structures are similar with the exception of pH-dependent variations at the ninth site that are detailed in Supplementary Fig. 3i,j. The representative U2AF^65^1,2L structure shown has the highest resolution and/or ribose nucleotide at the given site: (**a**) rU2 of structure iv; (**b**) rU3 of structure iii; (**c**) rU4 of structure i; (**d**) rU5 of structure iii; (**e**) rU6 of structure ii; (**f**) dU8 of structure iii; (**g**) dU9 of structure iii; (**h**) rC9 of structure iv. (**i**) Bar graph of apparent equilibrium affinities (*K*_A_) of the wild type (blue) and the indicated mutant (yellow) U2AF^65^1,2L proteins binding the AdML Py tract (5′-CCCUUUUUUUUCC-3′). The apparent equilibrium dissociation constants (*K*_D_) of the U2AF^65^1,2L mutant proteins are: wild type (WT), 35±6 nM; R227A, 166±2 nM; V254P, 137±10 nM; Q147A, 171±21 nM. The average *K*_A_ and s.e.m. for three independent titrations are plotted. The *P* values from two-tailed unpaired *t*-tests with Welch's correction are indicated as follows: **P<0.01; *P<0.05; NS, not significant, *P*>0.05. The average fitted fluorescence anisotropy RNA-binding curves are shown in Supplementary Fig. 4a–c.

The discovery of nine U2AF^65^-binding sites for contiguous Py-tract nucleotides was unexpected. Based on dU2AF^65^1,2 structures^14^,^15^,^20^, we originally hypothesized that the U2AF^65^ RRMs would bind the minimal seven nucleotides observed in these structures. Surprisingly, the RRM2 extension/inter-RRM linker contribute new central nucleotide-binding sites near the RRM1/RRM2 junction and the RRM1 extension recognizes the 3′-terminal nucleotide (Fig. 2c; Supplementary Movie 1). The U2AF^65^1,2L structures characterize ribose (r) nucleotides at all of the binding sites except the seventh and eighth deoxy-(d)U, which are likely to lack 2′-hydroxyl contacts based on the RNA-bound dU2AF^65^1,2 structure^15^. Qualitatively, a subset of the U2AF^65^1,2L-nucleotide-binding sites (sites 1–3 and 7–9) share similar locations to those of the dU2AF^65^1,2 structures (Supplementary Figs 2c,d and 3). Yet, only the U2AF^65^1,2L interactions at sites 1 and 7 are nearly identical to those of the dU2AF^65^1,2 structures (Supplementary Fig. 3a,f). In striking departures from prior partial views, the U2AF^65^1,2L structures reveal three unanticipated nucleotide-binding sites at the centre of the Py tract, as well as numerous new interactions that underlie cognate recognition of the Py tract (Fig. 3a–h).

### U2AF^65^ inter-RRM linker interacts with the Py tract

The U2AF^65^1,2L RRM2, the inter-RRM linker and RRM1 concomitantly recognize the three central nucleotides of the Py tract, which are likely to coordinate the conformational arrangement of these disparate portions of the protein. Residues in the C-terminal region of the U2AF^65^ inter-RRM linker comprise a centrally located binding site for the fifth nucleotide on the RRM2 surface and abutting the RRM1/RRM2 interface (Fig. 3d). The backbone amide of the linker V254 and the carbonyl of T252 engage in hydrogen bonds with the rU5-O4 and -N3H atoms. In the C-terminal β-strand of RRM1, the side chains of K225 and R227 donate additional hydrogen bonds to the rU5-O2 lone pair electrons. The C-terminal region of the inter-RRM linker also participates in the preceding rU4-binding site, where the V254 backbone carbonyl and D256 carboxylate position the K260 side chain to hydrogen bond with the rU4-O4 (Fig. 3c). Otherwise, the rU4 nucleotide packs against F304 in the signature ribonucleoprotein consensus motif (RNP)-2 of RRM2.

At the opposite side of the central fifth nucleotide, the sixth rU6 nucleotide is located at the inter-RRM1/RRM2 interface (Fig. 3e; Supplementary Movie 1). This nucleotide twists to face away from the U2AF^65^ linker and instead inserts the rU6-uracil into a sandwich between the β2/β3 loops of RRM1 and RRM2. The rU6 base edge is relatively solvent exposed; accordingly, the rU6 hydrogen bonds with U2AF^65^ are water mediated apart from a single direct interaction by the RRM1-N196 side chain.

We tested the contribution of the U2AF^65^1,2L interactions with the new central nucleotide to Py-tract affinity (Fig. 3i; Supplementary Fig. 4a,b). Mutagenesis of either V254 in the U2AF^65^ inter-RRM linker to proline or RRM1–R227 to alanine, which remove the hydrogen bond with the fifth uracil-O4 or -O2, reduced the affinities of U2AF^65^1,2L for the representative AdML Py tract by four- or five-fold, respectively. The energetic penalties due to these mutations (ΔΔ*G* 0.8–0.9 kcal mol^−1^) are consistent with the loss of each hydrogen bond with the rU5 base and support the relevance of the central nucleotide interactions observed in the U2AF^65^1,2L structures.

### U2AF^65^ RRM extensions interact with the Py tract

The N- and C-terminal extensions of the U2AF^65^ RRM1 and RRM2 directly contact the bound Py tract. Rather than interacting with a new 5′-terminal nucleotide as we had hypothesized, the C-terminal α-helix of RRM2 instead folds across one surface of rU3 in the third binding site (Fig. 3b). There, a salt bridge between the K340 side chain and nucleotide phosphate, as well as G338-base stacking and a hydrogen bond between the backbone amide of G338 and the rU3-O4, secure the RRM2 extension. Indirectly, the additional contacts with the third nucleotide shift the rU2 nucleotide in the second binding site closer to the C-terminal β-strand of RRM2. Consequently, the U2AF^65^1,2L-bound rU2-O4 and -N3H form dual hydrogen bonds with the K329 backbone atoms (Fig. 3a), rather than a single hydrogen bond with the K329 side chain as in the prior dU2AF^65^1,2 structure^15^ (Supplementary Fig. 3b).

At the N terminus, the α-helical extension of U2AF^65^ RRM1 positions the Q147 side chain to bridge the eighth and ninth nucleotides at the 3′ terminus of the Py tract (Fig. 3f–h). The Q147 residue participates in hydrogen bonds with the -N3H of the eighth uracil and -O2 of the ninth pyrimidine. The adjacent R146 guanidinium group donates hydrogen bonds to the 3′-terminal ribose-O2′ and O3′ atoms, where it could form a salt bridge with a phospho-diester group in the context of a longer pre-mRNA. Consistent with loss of a hydrogen bond with the ninth pyrimidine-O2 (ΔΔ*G* 1.0 kcal mol^−1^), mutation of the Q147 to an alanine reduced U2AF^65^1,2L affinity for the AdML Py tract by five-fold (Fig. 3i; Supplementary Fig. 4c). We compare U2AF^65^ interactions with uracil relative to cytosine pyrimidines at the ninth binding site in Fig. 3g,h and the Supplementary Discussion.

### Versatile primary sequence of the U2AF^65^ inter-RRM linker

**Figure 4:**
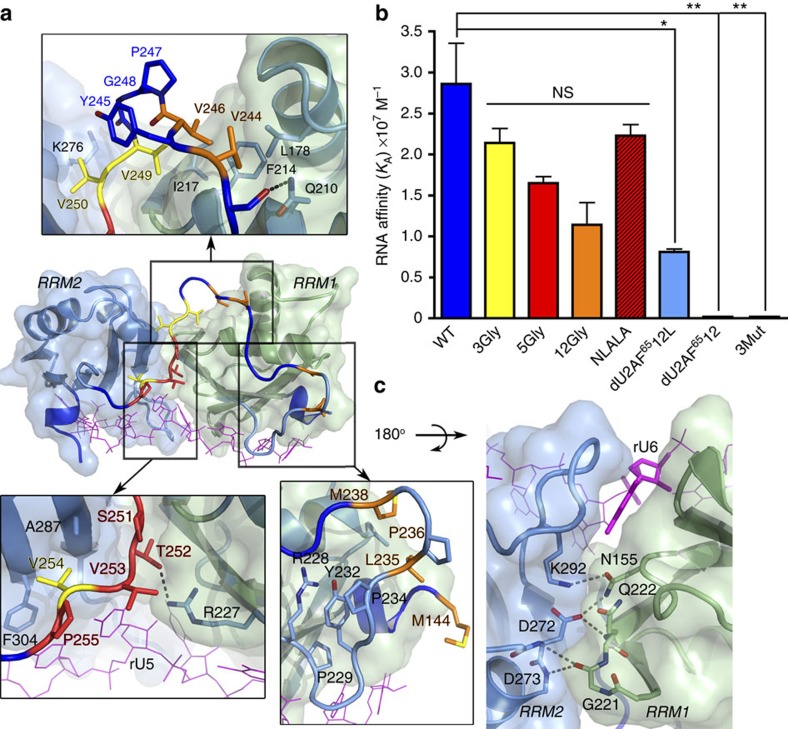
The U2AF^65^ linker/RRM and inter-RRM interactions. (**a**) Contacts of the U2AF^65^ inter-RRM linker with the RRMs. A semi-transparent space-filling surface is shown for the RRM1 (green) and RRM2 (light blue). Residues V249, V250, V254 (yellow) are mutated to V249G/V250G/V254G in the 3Gly mutant; residues S251, T252, V253, P255 (red) along with V254 are mutated to S251G/T252G/V253G/V254G/P255G in the 5Gly mutant or to S251N/T252L/V253A/V254L/P255A in the NLALA mutant; residues M144, L235, M238, V244, V246 (orange) along with V249, V250, S251, T252, V253, V254, P255 are mutated to M144G/L235G/M238G/V244G/V246G/V249G/ V250G/S251G/T252G/V253G/V254G/P255G in the 12Gly mutant. Other linker residues are coloured either dark blue for new residues in the U2AF^65^1,2L structure or light blue for the remaining inter-RRM residues. The central panel shows an overall view with stick diagrams for mutated residues; boxed regions are expanded to show the C-terminal (bottom left) and central linker regions (top) at the inter-RRM interfaces, and N-terminal linker region contacts with RRM1 (bottom right). (**b**) Bar graph of apparent equilibrium affinities (*K*_A_) for the AdML Py tract (5′-CCCUUUUUUUUCC-3′) of the wild-type (blue) U2AF^65^1,2L protein compared with mutations of the residues shown in **a**: 3Gly (yellow), 5Gly (red), NLALA (hatched red), 12Gly (orange) and the linker deletions dU2AF^65^1,2 in the minimal RRM1–RRM2 region (residues 148–237, 258–336) or dU2AF^65^1,2L (residues 141–237, 258–342). The apparent equilibrium dissociation constants (*K*_D_) of the U2AF^65^1,2L mutant proteins are: wild type (WT), 35±6 nM; 3Gly, 47±4 nM; 5Gly, 61±3 nM; 12Gly, 88±21 nM; NLALA, 45±3 nM; dU2AF^65^1,2L, 123±5 nM; dU2AF^65^1,2, 5000±100 nM; 3Mut, 5630±70 nM. The average *K*_A_ and s.e.m. for three independent titrations are plotted. The *P* values from two-tailed unpaired *t*-tests with Welch's correction are indicated as follows: ^**^P<0.01; *P<0.05; NS, not significant, *P*>0.05. The fitted fluorescence anisotropy RNA-binding curves are shown in Supplementary Fig. 4d–j. (**c**) Close view of the U2AF^65^ RRM1/RRM2 interface following a two-fold rotation about the *x*-axis relative to **a**.

The U2AF^65^1,2L structures reveal that the inter-RRM linker mediates an extensive interface with the second α-helix of RRM1, the β2/β3 strands of RRM2 and the N-terminal α-helical extension of RRM1. Altogether, the U2AF^65^ inter-RRM linker residues (R228–K260) bury 2,800 Å^2^ of surface area in the U2AF^65^1,2L holo-protein, suggestive of a cognate interface compared with 1,900 Å^2^ for a typical protein–protein complex^21^. The path of the linker initiates at P229 following the core RRM1 β-strand, in a kink that is positioned by intra-molecular stacking among the consecutive R228, Y232 and P234 side chains (Fig. 4a, lower right). A second kink at P236, coupled with respective packing of the L235 and M238 side chains on the N-terminal α-helical RRM1 extension and the core RRM1 α2-helix, reverses the direction of the inter-RRM linker towards the RRM1/RRM2 interface and away from the RNA-binding site. In the neighbouring apical region of the linker, the V244 and V246 side chains pack in a hydrophobic pocket between two α-helices of the core RRM1. The adjacent V249 and V250 are notable for their respective interactions that connect RRM1 and RRM2 at this distal interface from the RNA-binding site (Fig. 4a, top). A third kink stacks P247 and G248 with Y245 and re-orients the C-terminal region of the linker towards the RRM2 and bound RNA. At the RNA surface, the key V254 that recognizes the fifth uracil is secured via hydrophobic contacts between its side chain and the β-sheet surface of RRM2, chiefly the consensus RNP1-F304 residue that stacks with the fourth uracil (Fig. 4a, lower left). Few direct contacts are made between the remaining residues of the linker and the U2AF^65^ RRM2; instead, the C-terminal conformation of the linker appears primarily RNA mediated (Fig. 3c,d).

We investigated whether the observed contacts between the RRMs and linker were critical for RNA binding by structure-guided mutagenesis (Fig. 4b). We titrated these mutant U2AF^65^1,2L proteins into fluorescein-labelled AdML Py-tract RNA and fit the fluorescence anisotropy changes to obtain the apparent equilibrium affinities (Supplementary Fig. 4d–h). We introduced glycine substitutions to maximally reduce the buried surface area without directly interfering with its hydrogen bonds between backbone atoms and the base. First, we replaced V249 and V250 at the RRM1/RRM2 interface and V254 at the bound RNA site with glycine (3Gly). However, the resulting decrease in the AdML RNA affinity of the U2AF^65^1,2L-3Gly mutant relative to wild-type protein was not significant (Fig. 4b). In parallel, we replaced five linker residues (S251, T252, V253, V254 and P255) at the fifth nucleotide-binding site with glycines (5Gly) and also found that the RNA affinity of the U2AF^65^1,2L-5Gly mutant likewise decreased only slightly relative to wild-type protein. A more conservative substitution of these five residues (251–255) with an unrelated sequence capable of backbone-mediated hydrogen bonds (STVVP>NLALA) confirmed the subtle impact of this versatile inter-RRM sequence on affinity for the AdML Py tract. Finally, to ensure that these selective mutations were sufficient to disrupt the linker/RRM contacts, we substituted glycine for the majority of buried hydrophobic residues in the inter-RRM linker (including M144, L235, M238, V244, V246, V249, V250, S251, T252, V253, V254, P255; called 12Gly). Despite 12 concurrent mutations, the AdML RNA affinity of the U2AF^65^1,2L-12Gly variant was reduced by only three-fold relative to the unmodified protein (Fig. 4b), which is less than the penalty of the V254P mutation that disrupts the rU5 hydrogen bond (Fig. 3d,i).

To test the interplay of the U2AF^65^ inter-RRM linker with its N- and C-terminal RRM extensions, we constructed an internal linker deletion of 20-residues within the extended RNA-binding domain (dU2AF^65^1,2L). We found that the affinity of dU2AF^65^1,2L for the AdML RNA was significantly reduced relative to U2AF^65^1,2L (four-fold, Figs 1b and 4b; Supplementary Fig. 4i). Yet, it is well known that the linker deletion in the context of the minimal RRM1–RRM2 boundaries^15^,^16^,^22^ has no detectable effect on the RNA affinities of dU2AF^65^1,2 compared with U2AF^65^1,2 (refs 15, 16, 23; Figs 1b and 4b; Supplementary Fig. 4j). The U2AF^65^1,2L structures suggest that an extended conformation of the truncated dU2AF^65^1,2 inter-RRM linker would suffice to connect the U2AF^65^1,2L RRM1 C terminus to the N terminus of RRM2 (24 Å distance between U2AF^65^1,2L R227-Cα–H259-Cα atoms), which agrees with the greater RNA affinities of dU2AF^65^1,2 and U2AF^65^1,2 dual RRMs compared with the individual U2AF^65^ RRMs^23^. However, stretching of the truncated dU2AF^65^1,2L linker to connect the RRM termini is expected to disrupt its nucleotide interactions. Likewise, deletion of the N-terminal RRM1 extension in the shortened constructs would remove packing interactions that position the linker in a kinked turn following P229 (Fig. 4a), consistent with the lower RNA affinities of dU2AF^65^1,2L, dU2AF^65^1,2 and U2AF^65^1,2 compared with U2AF^65^1,2L.

To further test cooperation among the U2AF65 RRM extensions and inter-RRM linker for RNA recognition, we tested the impact of a triple Q147A/V254P/R227A mutation (U2AF^65^1,2L-3Mut) for RNA binding (Fig. 4b; Supplementary Fig. 4d). Notably, the Q147A/V254P/R227A mutation reduced the RNA affinity of the U2AF^65^1,2L-3Mut protein by 30-fold more than would be expected based on simple addition of the ΔΔG's for the single mutations. This difference indicates that the linearly distant regions of the U2AF^65^ primary sequence, including Q147 in the N-terminal RRM1 extension and R227/V254 in the N-/C-terminal linker regions at the fifth nucleotide site, cooperatively recognize the Py tract. Altogether, we conclude that the conformation of the U2AF^65^ inter-RRM linker is key for recognizing RNA and is positioned by the RRM extension but otherwise relatively independent of the side chain composition. The non-additive effects of the Q147A/V254P/R227A triple mutation, coupled with the context-dependent penalties of an internal U2AF^65^ linker deletion, highlights the importance of the structural interplay among the U2AF^65^ linker and the N- and C-terminal extensions flanking the core RRMs.

### Importance of U2AF^65^–RNA contacts for pre-mRNA splicing

**Figure 5:**
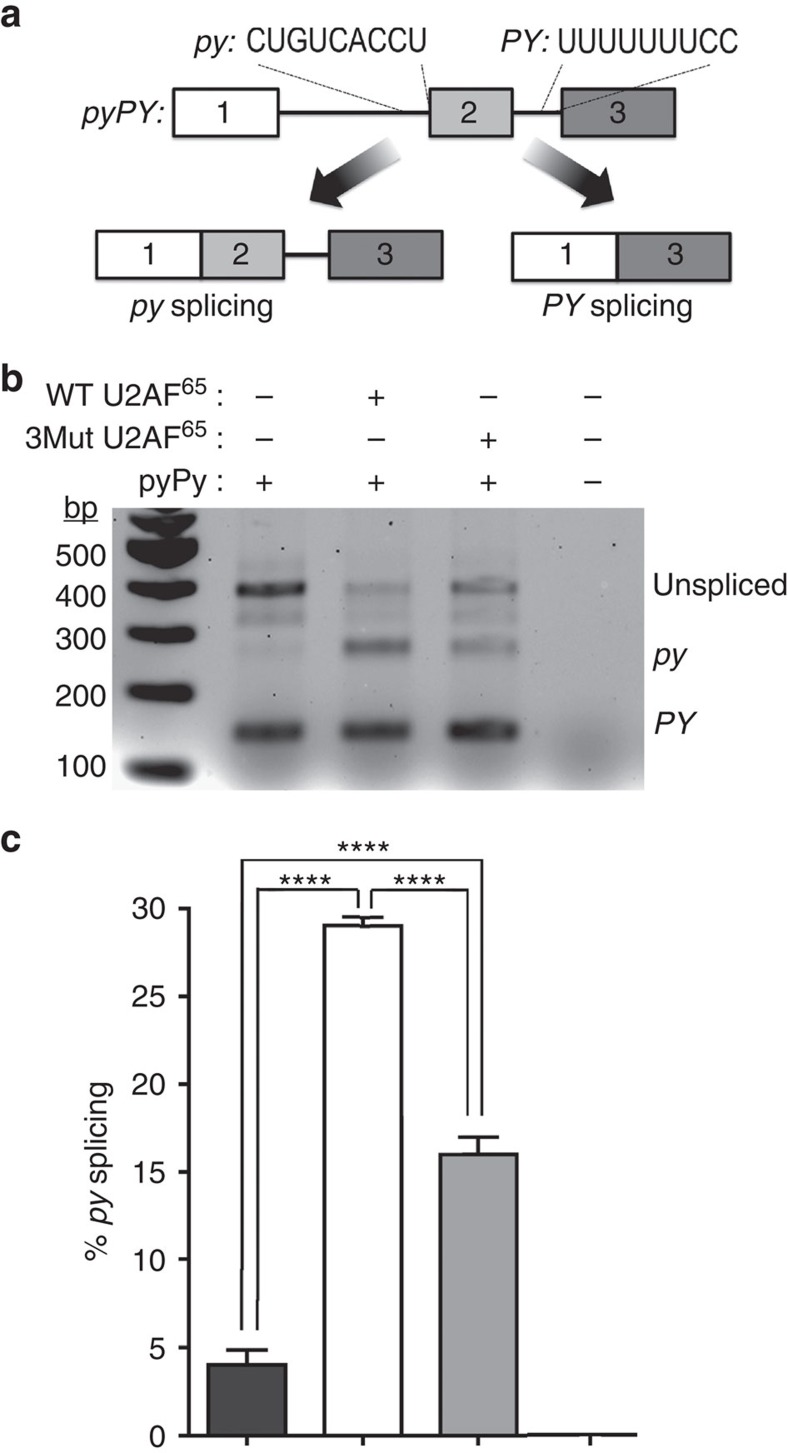
U2AF^65^ inter-domain residues are important for splicing a representative pre-mRNA substrate in human cells. (**a**) Schematic diagram of the *pyPY* reporter minigene construct comprising two alternative splice sites preceded by either the weak IgM Py tract (*py*) or the strong AdML Py tract (*PY*) (sequences inset). (**b**) Representative RT-PCR of *pyPY* transcripts from HEK293T cells co-transfected with constructs encoding the *pyPY* minigene and either wild-type (WT) U2AF^65^ or a triple U2AF^65^ mutant (3Mut) of Q147A, R227A and V254P residues. (**c**) A bar graph of the average percentage of the *py*-spliced mRNA relative to total detected *pyPY* transcripts (spliced and unspliced) for the corresponding gel lanes (black, no U2AF^65^ added; white, WT U2AF^65^; grey, 3Mut U2AF^65^). The average percentages and s.d.'s are given among four independent biological replicates. ^****^P<0.0001 for two-tailed unpaired *t*-test with Welch's correction. Protein overexpression and qRT-PCR results are shown in Supplementary Fig. 5.

We proceeded to test the importance of new U2AF^65^–Py-tract interactions for splicing of a model pre-mRNA substrate in a human cell line (Fig. 5; Supplementary Fig. 5). As a representative splicing substrate, we utilized a well-characterized minigene splicing reporter (called *pyPY*) comprising a weak (that is, degenerate, *py*) and strong (that is, U-rich, *PY*) polypyrimidine tracts preceding two alternative splice sites^24^ (Fig. 5a). When transfected into HEK293T cells containing only endogenous U2AF^65^, the *PY* splice site is used and the remaining transcript remains unspliced. When co-transfected with an expression plasmid for wild-type U2AF^65^, use of the *py* splice site significantly increases (by more than five-fold) and as documented^24^ converts a fraction of the unspliced to spliced transcript. The strong *PY* splice site is insensitive to added U2AF^65^, suggesting that endogenous U2AF^65^ levels are sufficient to saturate this site (Supplementary Fig. 5b). We introduced the triple mutation (V254P/R227A/Q147A) that significantly reduced U2AF^65^1,2L association with the Py tract (Fig. 4b) in the context of full-length U2AF^65^ (U2AF^65^-3Mut). Co-transfection of the U2AF^65^-3Mut with the *pyPY* splicing substrate significantly reduced splicing of the weak ‘*py*' splice site relative to wild-type U2AF^65^ (Fig. 5b,c). We conclude that the Py-tract interactions with these residues of the U2AF^65^ inter-RRM linker and RRM extensions are important for splicing as well as for binding a representative of the major U2-class of splice sites.

### Sparse inter-RRM contacts underlie apo-U2AF^65^ dynamics

The direct interface between U2AF^65^1,2L RRM1 and RRM2 is minor, burying 265 Å^2^ of solvent accessible surface area compared with 570 Å^2^ on average for a crystal packing interface^21^. A handful of inter-RRM hydrogen bonds are apparent between the side chains of RRM1-N155 and RRM2-K292, RRM1-N155 and RRM2-D272 as well as the backbone atoms of RRM1-G221 and RRM2-D273 (Fig. 4c). This minor U2AF^65^ RRM1/RRM2 interface, coupled with the versatile sequence of the inter-RRM linker, highlighted the potential role for inter-RRM conformational dynamics in U2AF^65^-splice site recognition. Paramagnetic resonance enhancement (PRE) measurements previously had suggested a predominant back-to-back, or ‘closed' conformation of the apo-U2AF^65^1,2 RRM1 and RRM2 in equilibrium with a minor ‘open' conformation resembling the RNA-bound inter-RRM arrangement^16^. Yet, small-angle X-ray scattering (SAXS) data indicated that both the minimal U2AF^65^1,2 and longer constructs comprise a highly diverse continuum of conformations in the absence of RNA that includes the ‘closed' and ‘open' conformations^25^,^26^. To complement the static portraits of U2AF^65^1,2L structure that we had determined by X-ray crystallography, we used smFRET to characterize the probability distribution functions and time dependence of U2AF^65^ inter-RRM conformational dynamics in solution.

**Figure 6:**
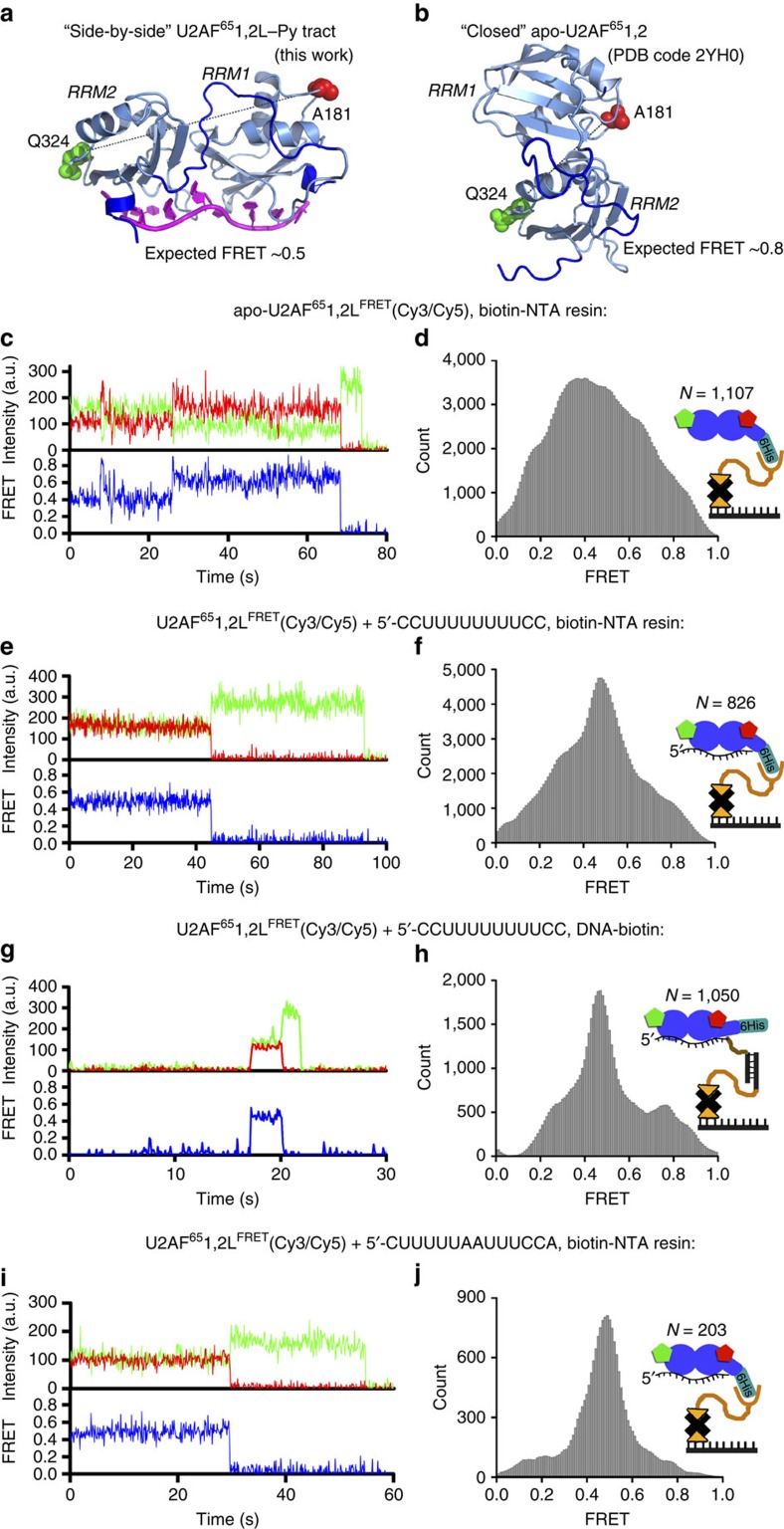
RNA binding stabilizes the side-by-side conformation of U2AF^65^ RRMs. (**a**,**b**) Views of FRET pairs chosen to follow the relative movement of RRM1 and RRM2 on the crystal structure of ‘side-by-side' U2AF^65^1,2L RRMs bound to a Py-tract oligonucleotide (**a**, representative structure *iv*) or ‘closed' NMR/PRE-based model of U2AF^65^1,2 (**b**, PDB ID 2YH0) in identical orientations of RRM2. The U2AF^65^1,2L^FRET^ proteins were doubly labelled at A181C/Q324C such that a mixture of Cy3/Cy5 fluorophores are expected to be present at each site. (**c**–**f**,**i**,**j**) The U2AF^65^1,2L^FRET^(Cy3/Cy5) protein was immobilized on the microscope slide via biotin-NTA/Ni^+2^ (orange line) on a neutravidin (black X)-biotin-PEG (orange triangle)-treated surface and imaged either in the absence of ligands (**c**,**d**), in the presence of 5 μM AdML Py-tract RNA (5′-CCUUUUUUUUCC-3′) (**e**,**f**), or in the presence of 10 μM adenosine-interrupted variant RNA (5′-CUUUUUAAUUUCCA-3′) (**i**,**j**). In **g** and **h**, the immobilization protocol was reversed. The untethered U2AF^65^1,2L^FRET^(Cy3/Cy5) protein (1 nM) was added to AdML RNA–polyethylene-glycol-linker–DNA oligonucleotide (10 nM), which was immobilized on the microscope slide by annealing with a complementary biotinyl-DNA oligonucleotide (black vertical line). Typical single-molecule FRET traces (**c**,**e**,**g**,**i**) show fluorescence intensities from Cy3 (green) and Cy5 (red) and the calculated apparent FRET efficiency (blue). Additional traces for untethered, RNA-bound U2AF^65^1,2L^FRET^(Cy3/Cy5) are shown in Supplementary Fig. 7c,d. Histograms (**d**,**f**,**h**,**j**) show the distribution of FRET values in RNA-free, slide-tethered U2AF^65^1,2L^FRET^(Cy3/Cy5) (**d**); AdML RNA-bound, slide-tethered U2AF^65^1,2L^FRET^(Cy3/Cy5) (**f**); AdML RNA-bound, untethered U2AF^65^1,2L^FRET^(Cy3/Cy5) (**h**) and adenosine-interrupted RNA-bound, slide-tethered U2AF^65^1,2L^FRET^(Cy3/Cy5) (**j**). *N* is the number of single-molecule traces compiled for each histogram.

The inter-RRM dynamics of U2AF^65^ were followed using FRET between fluorophores attached to RRM1 and RRM2 (Fig. 6a,b, Methods). The positions of single cysteine mutations for fluorophore attachment (A181C in RRM1 and Q324C in RRM2) were chosen based on inspection of the U2AF^65^1,2L structures and the ‘closed' model of apo-U2AF^65^1,2. Criteria included (i) residue locations that are distant from and hence not expected to interfere with the RRM/RNA or inter-RRM interfaces, (ii) inter-dye distances (50 Å for U2AF^65^1,2L–Py tract and 30 Å for the closed apo-model) that are expected to be near the Förster radius (*R*_o_) for the Cy3/Cy5 pair (56 Å)^27^, where changes in the efficiency of energy transfer are most sensitive to distance, and (iii) FRET efficiencies that are calculated to be significantly greater for the ‘closed' apo-model as opposed to the ‘open' RNA-bound structures (by ∼30%). The FRET efficiencies of either of these structurally characterized conformations also are expected to be significantly greater than elongated U2AF^65^ conformations that lack inter-RRM contacts.

Double-cysteine variant of U2AF^65^1,2 was modified with equimolar amount of Cy3 and Cy5. Only traces that showed single photobleaching events for both donor and acceptor dyes and anti-correlated changes in acceptor and donor fluorescence were included in smFRET data analysis. Hence, molecules that were conjugated to two donor or two acceptor fluorophores were excluded from analysis.

We first characterized the conformational dynamics spectrum of U2AF^65^ in the absence of RNA (Fig. 6c,d; Supplementary Fig. 7a,b). The double-labelled U2AF^65^1,2L^FRET^(Cy3/Cy5) protein was tethered to a slide via biotin-NTA/Ni^+2^ resin. Virtually no fluorescent molecules were detected in the absence of biotin-NTA/Ni^+2^, which demonstrates the absence of detectable non-specific binding of U2AF^65^1,2L^FRET^ to the slide. The FRET distribution histogram built from more than a thousand traces of U2AF^65^1,2L^FRET^(Cy3/Cy5) in the absence of ligand showed an extremely broad distribution centred at a FRET efficiency of ∼0.4 (Fig. 6d). Approximately 40% of the smFRET traces showed apparent transitions between multiple FRET values (for example, Fig. 6c). Despite the large width of the FRET-distribution histogram, the majority (80%) of traces that showed fluctuations sampled only two distinct FRET states (for example, Supplementary Fig. 7a). Approximately 70% of observed fluctuations were interchanges between the ∼0.65 and ∼0.45 FRET values (Supplementary Fig. 7b). We cannot exclude a possibility that tethering of U2AF^65^1,2L^FRET^(Cy3/Cy5) to the microscope slide introduces structural heterogeneity into the protein and, thus, contributes to the breadth of the FRET distribution histogram. However, the presence of repetitive fluctuations between particular FRET values supports the hypothesis that RNA-free U2AF^65^ samples several distinct conformations. This result is consistent with the broad ensembles of extended solution conformations that best fit the SAXS data collected for U2AF^65^1,2 as well as for a longer construct (residues 136–347)^25^. We conclude that weak contacts between the U2AF^65^ RRM1 and RRM2 permit dissociation of these RRMs in the absence of RNA.

### U2AF^65^ conformational selection and induced fit by bound RNA

We next used smFRET to probe the conformational selection of distinct inter-RRM arrangements following association of U2AF^65^ with the AdML Py-tract prototype. Addition of the AdML RNA to tethered U2AF^65^1,2L^FRET^(Cy3/Cy5) selectively increases a fraction of molecules showing an ∼0.45 apparent FRET efficiency, suggesting that RNA binding stabilizes a single conformation, which corresponds to the 0.45 FRET state (Fig. 6e,f). To assess the possible contributions of RNA-free conformations of U2AF^65^ and/or structural heterogeneity introduced by tethering of U2AF^65^1,2L^FRET^(Cy3/Cy5) to the slide to the observed distribution of FRET values, we reversed the immobilization scheme. We tethered the AdML RNA to the slide via a biotinylated oligonucleotide DNA handle and added U2AF^65^1,2L^FRET^(Cy3/Cy5) in the absence of biotin-NTA resin (Fig. 6g,h; Supplementary Fig. 7c–g). A 0.45 FRET value was again predominant, indicating a similar RNA-bound conformation and structural dynamics for the untethered and tethered U2AF^65^1,2L^FRET^(Cy3/Cy5).

We examined the effect on U2AF^65^1,2L conformations of purine interruptions that often occur in relatively degenerate human Py tracts^2^. We introduced an rArA purine dinucleotide within a variant of the AdML Py tract (detailed in Methods). Insertion of adenine nucleotides decreased binding affinity of U2AF65 to RNA by approximately five-fold. Nevertheless, in the presence of saturating concentrations of rArA-interrupted RNA slide-tethered U2AF651,2LFRET(Cy3/Cy5) showed a prevalent ∼0.45 apparent FRET value (Fig. 6i,j), which was also predominant in the presence of continuous Py tract. Therefore, RRM1-to-RRM2 distance remains similar regardless of whether U2AF^65^ is bound to interrupted or continuous Py tract.

The inter-fluorophore distances derived from the observed 0.45 FRET state agree with the distances between the α-carbon atoms of the respective residues in the crystal structures of U2AF^65^1,2L bound to Py-tract oligonucleotides. It should be noted that inferring distances from FRET values is prone to significant error because of uncertainties in the determination of fluorophore orientation factor *κ*^2^ and Förster radius *R*_0_, the parameters used in distance calculations^28^. Nevertheless, the predominant 0.45 FRET state in the presence of RNA agrees with the Py-tract-bound crystal structure of U2AF^65^1,2L.

Importantly, the majority of traces (∼70%) of U2AF^65^1,2L^FRET^(Cy3/Cy5) bound to the slide-tethered RNA lacked FRET fluctuations and predominately exhibited a ∼0.45 FRET value (for example, Fig. 6g). The remaining ∼30% of traces for U2AF^65^1,2L^FRET^(Cy3/Cy5) bound to the slide-tethered RNA showed fluctuations between distinct FRET values. The majority of traces that show fluctuations began at high (0.65–0.8) FRET value and transitioned to a ∼0.45 FRET value (Supplementary Fig. 7c–g). Hidden Markov modelling analysis of smFRET traces suggests that RNA-bound U2AF^65^1,2L can sample at least two other conformations corresponding to ∼0.7–0.8 and ∼0.3 FRET values in addition to the predominant conformation corresponding to the 0.45 FRET state. Although a compact conformation (or multiple conformations) of U2AF^65^1,2L corresponding to ∼0.7–0.8 FRET values can bind RNA, on RNA binding, these compact conformations of U2AF^65^1,2L transition into a more stable structural state that corresponds to ∼0.45 FRET value and is likely similar to the side-by-side inter-RRM-arrangement of the U2AF^65^1,2L crystal structures. Thus, the sequence of structural rearrangements of U2AF^65^ observed in smFRET traces (Supplementary Fig. 7c–g) suggests that a ‘conformational selection' mechanism of Py-tract recognition (that is, RNA ligand stabilization of a pre-configured U2AF^65^ conformation) is complemented by ‘induced fit' (that is, RNA-induced rearrangement of the U2AF^65^ RRMs to achieve the final ‘side-by-side' conformation), as discussed below.

## Discussion

The U2AF^65^ structures and analyses presented here represent a successful step towards defining a molecular map of the 3′ splice site. Several observations indicate that the numerous intramolecular contacts, here revealed among the inter-RRM linker and RRM1, RRM2, and the N-terminal RRM1 extension, synergistically coordinate U2AF^65^–Py-tract recognition. Truncation of U2AF^65^ to the core RRM1–RRM2 region reduces its RNA affinity by 100-fold. Likewise, deletion of 20 inter-RRM linker residues significantly reduces U2AF^65^–RNA binding only when introduced in the context of the longer U2AF^65^1,2L construct comprising the RRM extensions, which in turn position the linker for RNA interactions. Notably, a triple mutation of three residues (V254P, Q147A and R227A) in the respective inter-RRM linker, N- and C-terminal extensions non-additively reduce RNA binding by 150-fold. Altogether, these data indicate that interactions among the U2AF^65^ RRM1/RRM2, inter-RRM linker, N-and C-terminal extensions are mutually inter-dependent for cognate Py-tract recognition. The implications of this finding for U2AF^65^ conservation and Py-tract recognition are detailed in the Supplementary Discussion.

**Figure 7:**
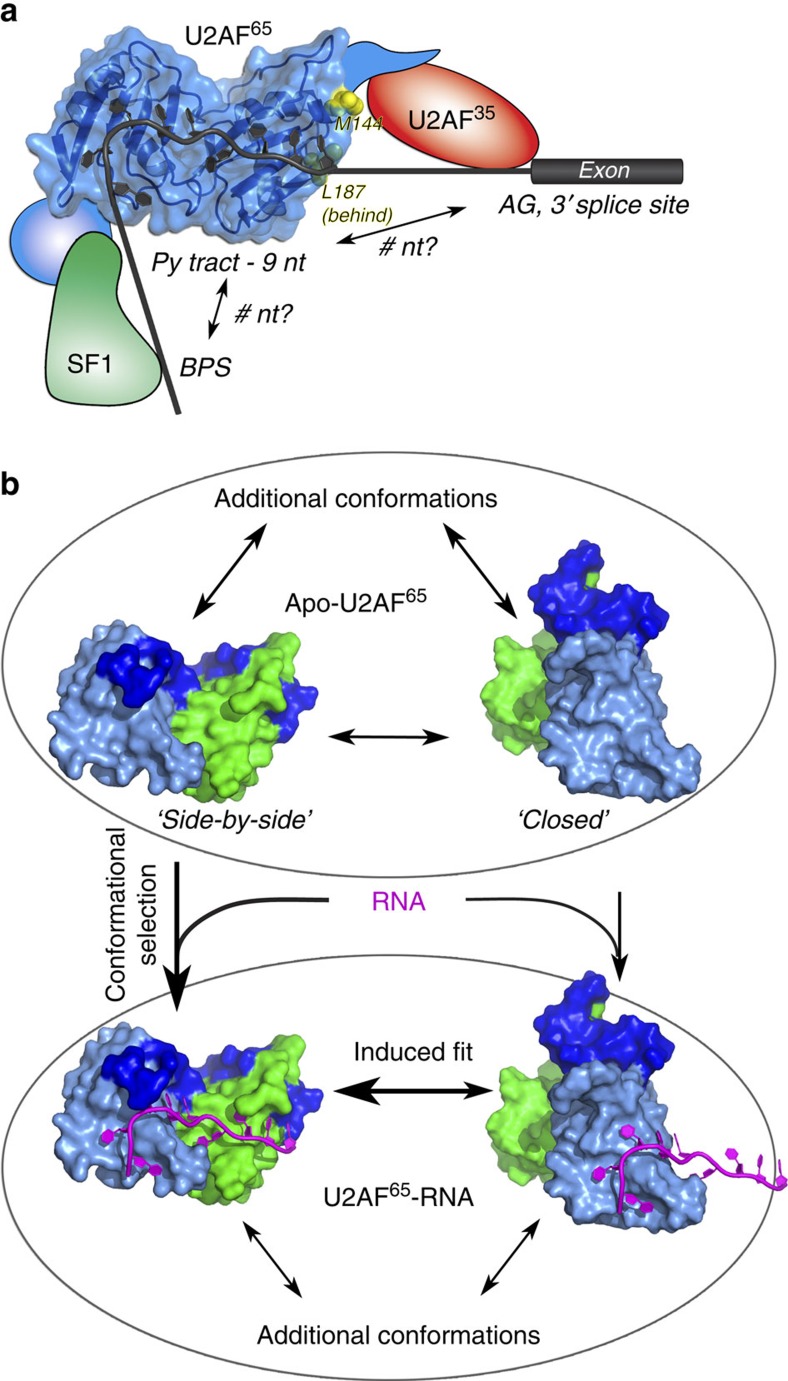
Schematic models of U2AF^65^ recognizing the Py tract. (**a**) Diagram of the U2AF^65^, SF1 and U2AF^35^ splicing factors bound to the consensus elements of the 3′ splice site. A surface representation of U2AF^65^1,2L is shown bound to nine nucleotides (nt); the relative distances and juxtaposition of the branch point sequence (BPS) and consensus AG dinucleotide at the 3′ splice site are unknown. MDS-relevant mutated residues of U2AF^65^ are shown as yellow spheres (L187 and M144). (**b**) Following binding to the Py-tract RNA, a conformation corresponding to high FRET and consistent with the ‘closed', back-to-back apo-U2AF^65^ model resulting from PRE/NMR characterization (PDB ID 2YH0) often transitions to a conformation corresponding to ∼0.45 FRET value, which is consistent with ‘open', side-by-side RRMs such as the U2AF^65^1,2L crystal structures. Alternatively, a conformation of U2AF^65^ corresponding to ∼0.45 FRET value can directly bind to RNA; RNA binding stabilizes the ‘open', side-by-side conformation and thus shifts the U2AF^65^ population towards the ∼0.45 FRET value. RRM1, green; RRM2, pale blue; RRM extensions/linker, blue.

Recently, high-throughput sequencing studies have shown that somatic mutations in pre-mRNA splicing factors occur in the majority of patients with myelodysplastic syndrome (MDS)^29^. MDS-relevant mutations are common in the small U2AF subunit (U2AF^35^, or U2AF1), yet such mutations are rare in the large U2AF^65^ subunit (also called U2AF2)—possibly due to the selective versus nearly universal requirements of these factors for splicing. A confirmed somatic mutation of U2AF^65^ in patients with MDS, L187V^30^, is located on a solvent-exposed surface of RRM1 that is distinct from the RNA interface (Fig. 7a). This L187 surface is oriented towards the N terminus of the U2AF^65^1,2L construct, where it is expected to abut the U2AF^35^-binding site in the context of the full-length U2AF heterodimer. Likewise, an unconfirmed M144I mutation reported by the same group corresponds to the N-terminal residue of U2AF^65^1,2L, which is separated by only ∼20 residues from the U2AF^35^-binding site^31^. As such, we suggest that the MDS-relevant U2AF^65^ mutations contribute to MDS progression indirectly, by destabilizing a relevant conformation of the conjoined U2AF^35^ subunit rather than affecting U2AF^65^ functions in RNA binding or spliceosome recruitment *per se.*

Our smFRET results agree with prior NMR/PRE evidence for multi-domain conformational selection^16^ as one mechanistic basis for U2AF^65^–RNA association (Fig. 7b). The ‘induced fit' versus ‘conformational selection' models are the prevailing views of the mechanisms underlying bio-molecular interactions (reviewed in ref. 32). In the former, ligand binding promotes a subsequent conformational change in the protein, whereas in the latter, the ligand selects a protein conformation from a pre-existing ensemble and thereby shifts the population towards that state. An ∼0.45 FRET value is likely to correspond to the U2AF^65^ conformation visualized in our U2AF^65^1,2L crystal structures, in which the RRM1 and RRM2 bind side-by-side to the Py-tract oligonucleotide. The lesser 0.65–0.8 and 0.2–0.3 FRET values in the untethered U2AF^65^1,2LFRET(Cy3/Cy5) experiment could correspond to respective variants of the ‘closed', back-to-back U2AF^65^ conformations characterized by NMR/PRE data^16^, or to extended U2AF^65^ conformations, in which the intramolecular RRM1/RRM2 interactions have dissociated the protein is bound to RNA via single RRMs. An increased prevalence of the ∼0.45 FRET value following U2AF^65^–RNA binding, coupled with the apparent absence of transitions in many ∼0.45-value single molecule traces (for example, Fig. 6e), suggests a population shift in which RNA binds to (and draws the equilibrium towards) a pre-configured inter-RRM proximity that most often corresponds to the ∼0.45 FRET value.

Notably, our smFRET results reveal that U2AF^65^–Py-tract recognition can be characterized by an ‘extended conformational selection' model (Fig. 7b). In this recent model for macromolecular interactions^32^,^33^, the pure ‘conformational selection' and ‘induced fit' scenarios represent the limits of a mechanistic spectrum and may compete or occur sequentially. Examples of ‘extended conformational selection' during ligand binding have been characterized for a growing number of macromolecules (for example, adenylate kinase^34^,^35^, LAO-binding protein^36^, poly-ubiquitin^37^, maltose-binding protein^38^ and the preQ1 riboswitch^39^, among others). Here, the majority of changes in smFRET traces for U2AF^65^1,2L^FRET^(Cy3/Cy5) bound to slide-tethered RNA began at high (0.65–0.8) FRET value and transition to the predominant 0.45 FRET value (Supplementary Fig. 7c–g). These transitions could correspond to rearrangement from the ‘closed' NMR/PRE-based U2AF^65^ conformation in which the RNA-binding surface of only a single RRM is exposed and available for RNA binding^16^, to the structural state seen in the side-by-side, RNA-bound crystal structure. As such, the smFRET approach reconciles prior inconsistencies between two major conformations that were detected by NMR/PRE experiments^16^ and a broad ensemble of diverse inter-RRM arrangements that fit the SAXS data for the apo-protein^25^,^26^. Similar interdisciplinary structural approaches are likely to illuminate whether similar mechanistic bases for RNA binding are widespread among other members of the vast multi-RRM family.

The finding that U2AF^65^ recognizes a nine base pair Py tract contributes to an elusive ‘code' for predicting splicing patterns from primary sequences in the post-genomic era (reviewed in ref. 40). Based on (i) similar RNA affinities of U2AF^65^ and U2AF^65^1,2L, (ii) indistinguishable conformations among four U2AF^65^1,2L structures in two different crystal packing arrangements and (iii) penalties of structure-guided mutations in RNA binding and splicing assays, we suggest that the extended inter-RRM regions of the U2AF^65^1,2L structures underlie cognate Py-tract recognition by the full-length U2AF^65^ protein. Further research will be needed to understand the roles of SF1 and U2AF^35^ subunits in the conformational equilibria underlying U2AF^65^ association with Py tracts. Moreover, structural differences among U2AF^65^ homologues and paralogues may regulate splice site selection. Ultimately, these guidelines will assist the identification of 3′ splice sites and the relationship of disease-causing mutations to penalties for U2AF^65^ association.

## Methods

### Protein expression and purification

For crystallization and RNA-binding experiments, human U2AF^65^1,2L (residues 141–342 of NCBI RefSeq NP_009210) was expressed in *Escherichia coli* strain BL21 Rosetta-2 as a GST-fusion protein in the vector pGEX6P-2 and purified by glutathione affinity, followed by anion exchange and gel filtration chromatography. The GST-tagged protein was bound to a GSTrap column (GE Healthcare) in 1 M NaCl, 25 mM HEPES, pH 7.4 and eluted using 150 mM NaCl, 100 mM Tris, pH 8 containing 10 mM glutathione. The GST tag was cleaved from the protein by treatment with PreScission Protease during dialysis against a buffer containing 100 mM NaCl, 25 mM HEPES, pH 8, 5% (v/v) glycerol, 5 mM DTT, 0.25 mM EDTA and 0.1 mM PMSF. Cleaved GST was separated from the U2AF^65^1,2L by subtractive glutathione affinity chromatography in 100 mM NaCl, 25 mM Tris, pH 8, 0.2 mM TCEP followed by subtractive anion-exchange chromatography with a HiTrap Q column (GE Healthcare). The final purification step was size-exclusion chromatography on a Superdex-75 prep-grade column (GE Healthcare) that had been previously equilibrated with 100 mM NaCl, 15 mM HEPES, pH 6.8, 0.2 mM tris(2-carboxy-ethyl)phosphine (TCEP). The purified U2AF^65^1,2L was concentrated using a Vivaspin 15 R (Sartorius) centrifugal concentrator with 10 kDa MWCO, and the protein concentration was estimated using the calculated extinction coefficient of 8,940 M^−1^cm^−1^ and absorbance at 280 nm. Shorter constructs (U2AF^65^1,2, residues 148–336; dU2AF^65^1,2, residues 148–237, 258–336; dU2AF^65^1,2L, residues 141–237, 258–342) (Fig. 1a) and individual U2AF^65^1,2L Q147A, R227A, V254P mutants used for RNA-binding experiments were purified similarly.

For comparative RNA-binding experiments, full-length human U2AF^65^ (residues 1–475) and the U2AF^35^-UHM (U2AF homology motif; residues 43–146, NCBI RefSeq NP_006749) initially were expressed and purified separately as GST fusion proteins. Following GST cleavage and ion-exchange chromatography (SP-HiTrap and Q-HiTrap, respectively), U2AF^65^ was combined with slight excess U2AF^35^-UHM (in stoichiometric ratio of 1:1.2) and dialysed overnight. The final U2AF heterodimer was purified by size-exclusion chromatography using a Superdex-200 prep-grade column (GE Healthcare) pre-equilibrated with 150 mM NaCl, 25 mM HEPES, pH 6.8, 0.2 mM TCEP. Representative purified U2AF^65^1,2L and U2AF65–U2AF^35^-UHM proteins are shown in Supplementary Fig. 1a.

### Oligonucleotide preparation

High-performance liquid chromatography-purified oligonucleotides (sequences shown in Supplementary Fig. 2a) were purchased for crystallization (Integrated DNA Technologies, Inc.). The lyophilized oligonucleotides were diluted in gel filtration buffer for crystallization experiments. The 5′-fluorescein (Fl)-labelled RNAs (AdML: 5′-Fl-CCCUUUUUUUUCC-3′, Py tract of the AdML splicing substrate; 5′-4rU: 5′-Fl-CCUUUUCCCCCCC-3′; 3′-4rU: 5′-Fl-CCCCCCCUUUUCC-3′) for RNA-binding experiments (Dharmacon Research, Inc., Thermo Scientific) was deprotected according to the manufacturer's protocol, vacuum dried and resuspended in nuclease-free water. RNA and RNA–DNA concentrations were calculated using the calculated molar extinction coefficients^41^ and absorbance at 260 nm.

### Fluorescence anisotropy RNA-binding experiments

For RNA-binding experiments, purified proteins and RNA were diluted separately >100-fold in binding buffer (100 mM NaCl, 15 mM HEPES, pH 6.8, 0.2 mM TCEP, 0.1 U μl^−1^ Superase-In (Ambion Life Technologies)). The final RNA concentration in the cuvette was 30 nM. Volume changes during addition of the protein were <10% to minimize dilution effects. The fluorescence anisotropy changes during titration were measured using a FluoroMax-3 spectrophotometer temperature controlled by a circulating water bath at 23 °C. Samples were excited at 490 nm and emission intensities recorded at 520 nm with a slit width of 5 nm. The titrations were repeated three times in succession. Each titration was fit with Graphpad Prism v4.0 to obtain the apparent equilibrium dissociation constant (*K*_D_)^22^. The apparent equilibrium affinities (*K*_A_) are the reciprocal of the *K*_D_. The average *K*_D_'s or *K*_A_'s and s.e.m. among the three replicates were calculated using Excel and are reported in Figs 3 and 4; Supplementary Figs 1 and 4. The *P* values from a two-tailed unpaired *t*-test were calculated using Graphpad Prism v4.0.

### Transfection, immunoblotting and RT-PCR analyses

For transfection experiments, the full-length human U2AF^65^ cDNA in pCMV6-XL5 (Origene Tech. Inc., clone ID BC008740) was used (WT U2AF^65^) and in parallel mutated to encode the Q147A/R227A/V254P triple-mutant protein (Mut U2AF^65^). The *pyPY* minigene was a gift from M. Carmo-Fonseca (University of Lisbon, Portugal)^24^. HEK293T cells (kindly provided by Dr Lata Balakrishnan, originally purchased from ATCC, cat. no. CRL3216) were seeded into 12-well plates (2–4 × 10^5^ cells per well) and grown as monolayers in MEM (Gibco Life Technologies) supplemented with 10% (v/v) of heat-inactivated fetal bovine serum, 1% (v/v) L-glutamine and 1% (v/v) penicillin–streptomycin. After 1 day, the cells were transiently transfected with either 0.5 μg of *pyPY* plasmid or a mixture of 0.5 μg of U2AF^65^ variant and 0.5 μg of *pyPY* plasmid per well using appropriately adjusted Lipofectamine 2000 (Invitrogen Life Technologies) ratio according to the manufacturer's instructions.

For immunoblots of WT U2AF^65^ and Mut U2AF^65^ expression levels (Supplementary Fig. 5a), transfected or control cells were lysed in radioimmunoprecipitation assay buffer with proteinase and kinase inhibitors. Total protein (20 μg) was separated by SDS–PAGE, and transferred onto polyvinylidene difluoride membranes (Millipore Corp., Billerica, MA, USA) and immunoblotted using mouse monoclonal antibodies directed against U2AF^65^ (ref. 42) (MC3, cat. no. U4758 Sigma-Aldrich at 1:500 dilution) or as a control for comparison, GAPDH (glyceraldehyde-3-phosphate dehydrogenase; monoclonal clone 71.1, cat. no. G8795 Sigma-Aldrich at 1:5,000 dilution). Immunoblots were developed using anti-mouse horseradish peroxidase-conjugates (cat. no. U4758 Sigma-Aldrich, Co. at 1:2,500 or 1:10,000 dilutions for GAPDH and U2AF^65^, respectively) and detected using SuperSignal WestPico chemi-luminescent substrate (Pierce Thermo Scientific Inc.). Blots were imaged using a IS4000MM system (Carestream, Rochester, NY, USA). For size analysis, fluorescent images of the BioRad Precision Plus Dual Color Standards were overlaid directly.

For reverse transcription PCR (RT-PCR), the total RNA was isolated 2 days post transfection using the Cells-to-cDNA II kit (Ambion Life Technologies). The RT-PCR reaction comprised 35 cycles (94 °C per 60 s—60 °C per 50 s—72 °C per 60 s) with forward (5′-TGAGGGGAGGTGAATGAGGAG-3′) and reverse (5′-TCCACTGGAAAGACCGCGAAG-3′) primers for the *pyPY* product or forward (5′-CATGTTCGTCATGGGTGTGAACCA-3′) and reverse (5′-ATGGCATGGACTGTGGTCATGAGT-3′) primers for a GAPDH control. The RT-PCR products were separated by 2% agarose gel electrophoresis and stained with ethidium bromide. The percentages of splice site use were calculated from the background corrected intensities *I* using the formula 100% × *I*(*py*)*/*[*I*(*py*)*+I*(*PY*)+*I*(unspliced)] for *py* spliced (Fig. 5b,c) or 100% × *I*(*PY*)*/*[*I*(*py*)*+I*(*PY*)+*I*(unspliced)] for *PY* spliced (Supplementary Fig. 5b). The band intensities of four independent biological replicates were measured using ImageQuant software.

### Crystallization, data collection and structure determination

Before crystallization, the purified U2AF^65^1,2L and given oligonucleotide were mixed to achieve respective final concentrations of 1.0 and 1.1 mM and incubated on ice for 20–30 min. For each oligonucleotide, sparse matrix screens of the Jancarik and Kim Crystal Screen^43^(in hanging drop format; Hampton Research, Corp.) and JCSG-Plus (in sitting drop format; Molecular Dimensions) were used to identify initial crystallization conditions, which were obtained from the latter screen and further optimized in hanging drop format. In optimized crystallization experiments, a mixture of sample and reservoir solution (1.2:1 μl) was equilibrated against 700 μl reservoir solution at 4 °C.

The oligonucleotide sequences were optimized and the structures were determined as follows: in addition to the previously characterized dU2AF^65^1,2-binding sites for seven nucleotides, the new terminal residues of the U2AF^65^1,2L construct were presumed to contact an additional nucleotide and the crystal packing of a central nucleotide between the RRM1/RRM2 of dU2AF^65^1,2 was presumed to represent one nucleotide. Also considering the known proclivity for deoxy(d)U to co-crystallize with dU2AF^65^1,2 (ref. 44) and for 5-bromo-dU (5BrdU) to bind a given site of dU2AF^65^1,2 (ref. 14), we initially designed two 9-mer oligonucleotides (5′-ribose (r)UrUrUrUrU(5BrdU)dUrUrU and 5′-rUrUrUdUdU(5BrdU)dUrUrU) and screened for co-crystallization with U2AF^65^1,2L. The former oligonucleotide failed to produce crystals in these screens. The latter oligonucleotide comprising central dU nucleotides produced diffracting crystals, which were frozen directly from a reservoir comprising 100 mM phosphate–citrate buffer pH 4.2, 40% Peg 300. The structure determined by molecular replacement using Phenix^45^ with a data set collected at beamline (BL) 12-2 of the Stanford Synchrotron Radiation Lightsource (SSRL; Menlo Park, CA, USA) (Table 1). The search models comprising each of the individual RRMs bound to two nucleotides were derived from the dU2AF^65^1,2 structure (PDB ID 2G4B) (translation function *Z*-score equivalent 12.9, log-likelihood gain 528). For comparison, searches with the NMR structure (PDB ID 2YH1) as a search model failed to find a solution. The initial structure revealed a greater number of central nucleotide-binding sites than expected. The oligonucleotide binding register had slipped to place the BrdU in the preferred site, leave the 5′ terminal-binding sites empty, and the terminal nucleotide unbound and disordered. Subsequent oligonucleotides were designed to place BrdU in the preferred site, fill the unoccupied 5′ terminal sites, capture rU at the central sites, and compare rC at the terminal site.

The U2AF^65^1,2L protein co-crystals with oligonucleotide 5′-phosphorylated (P)-rUrUdUdUrUdU(BrdU)dU were obtained using a reservoir of 200 mM LiCl, 100 mM sodium citrate pH 4.0, 8% (w/v) polyethylene glycol (PEG) 6,000, 10% (v/v) PEG 300, 10% (v/v) dioxane with 0.1 μl of *N*,*N*-bis[3-(D-gluconamido)propyl]deoxy-cholamide (deoxy-BigCHAP) (14 mM) added to the hanging drop and cryoprotected by sequential layering with reservoir solution supplemented with increasing PEG 300 to a final concentration of 26%. Co-crystals with either 5′-(P)rUrUdUrUrU(BrdU)dUdU or 5′-(P)rUrUrUdUrUrU(BrdU)dUrC were obtained from 1 M succinate, 100 mM HEPES, pH 7.0, 1–3% (w/v) PEG monomethylether 2,000. The former was cryoprotected by coating with a 1:1 (v/v) mixture of silicon oil and Paratone-N and the latter by sequential transfer to 21% (v/v) glycerol. Data sets for flash-cooled crystals were collected at 100 K using remote access to SSRL BL12-2. Structures were determined by molecular replacement using the initial U2AF^65^1,2L/rUrUrUdUdU(BrdU)dUrUrU structure as a search model. Consistent sets of free-R reflections were maintained (6% of the total reflections). Models were built using COOT^46^ and refined with PHENIX^45^. No non-glycine/non-proline residues were found in the disallowed regions of the Ramachandran plots. Clash scores and Molprobity scores calculated using the program Molprobity^47^ were above average. Structure illustrations were prepared using PYMOL^48^. Crystallographic data and refinement statistics are given in Table 1.

### Sample preparation for single-molecule FRET

The U2AF^65^1,2L^FRET^ construct used for smFRET comprises the six histidine and T7 tags from the pET28a vector (Merck), a GGGS linker and U2AF^65^ residues 113–343. The single cysteine of human U2AF^65^ was replaced by alanine (C305A), which is a natural amino-acid variation among U2AF^65^ homologues. Single A181C and Q324C mutations were introduced in each RRM for fluorophore attachment at residues that were carefully selected to meet experimental criteria described in the Results. The U2AF^65^1,2L^FRET^ was purified by the same method as described above for U2AF^65^1,2L protein and binds RNA with similar affinity as U2AF^65^1,2L (Supplementary Fig. 6a). Before labelling, the purified U2AF^65^1,2L^FRET^ protein was incubated with 10 mM DTT on ice for 30 min and then buffer exchanged into Labelling Buffer (100 mM NaCl, 25 mM HEPES pH 7.0, 5 mM EDTA, 0.5 mM tris(2-carboxy-ethyl)phosphine (TCEP)) using Zeba Spin Desalting Columns 7K MWCO (Pierce, ThermoFisher Scientific). To initiate the labelling reaction, 4 μl each of cyanine (Cy)3-Maleimide and Cy5-Maleimide (Combinix, Inc.) stock solutions (10 mM in DMSO) were pre-mixed (total volume 8 μl) and then added to 200 μl of 20 μM protein (final 20:1 molar ratio of dye:protein). The labelling reaction was incubated at room temperature in the dark for 2 h and then quenched by the addition of 10 mM DTT. The labelled protein was separated from excess dye using a Zeba Spin Desalting Column followed by size exclusion chromatography using a pre-packed Superdex-75 10/300 GL (GE Healthcare) column in Labelling Buffer. Our previous experience of conjugating cysteines with maleimide derivatives of fluorophores and suggests that nonspecific modification of aminogroups of proteins with fluorescent dyes under the employed experimental conditions is negligible. Consistent with specific labelling of A181C and Q324C, the labelling efficiencies were ∼60% each for Cy3 and Cy5 as estimated using the dye extinction coefficients (*ɛ*^Cy3^=150,000 M^−1^ cm^−1^ at 550 nm, *ɛ*^Cy5^=170,000 M^−1^ cm^−1^ at 650 nm) and the calculated extinction coefficient of the U2AF^65^1,2L^FRET^ protein (*ɛ*^prot^=8,940 M^−1^ cm^−1^ at 280 nm), and correcting for the absorbance (*A*) of the dyes at 280 nm (GE Healthcare, Amersham CyDye Maleimide product booklet):


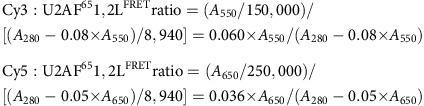


For smFRET experiments with a ‘strong', homogeneous Py tract, we used the prototypical AdML sequence (5′-CCUUUUUUUUCC-3′). To investigate the inter-RRM separation in the presence of a ‘weak' Py tract interrupted by purines, we compared the U2AF^65^1,2L affinity for a purine-interrupted Py tract comprising an rUrUrUrUrU tract that is expected to bind U2AF^65^ RRM2/inter-RRM linker, a central rArA and an rUrUrUrCrC tract that is expected to bind RRM1. The tandem purines represent a compromise between significant inhibition of U2AF^65^ binding by longer A interruptions^16^ and an approximately five-fold penalty for the rArA mutation in the AdML Py tract (Supplementary Fig. 6b,c). To maintain avidity and provide flanking phosphoryl groups in case of inter-RRM adjustment, we included the 5′-C and 3′-A of parent AdML sequence, which are respective low-affinity nucleotides for binding RRM2 and RRM1 (ref. 14), in the final rArA-interrupted RNA oligonucleotide (5′-rCrUrUrUrUrUrArArUrUrUrCrCrA-3′).

For the reversed immobilization of RNA via a complementary biotinyl-DNA primer experiment, the AdML Py-tract RNA was extended to include the DNA counterpart of downstream AdML intron/exon sequences that were complementary to the biotinyl-DNA primer. To increase separation from the slide surface, a hexaethylene glycol linker (18PEG) was inserted between the AdML Py-tract RNA and the tethered DNA duplex. The tethered oligonucleotide sequences included: 5′-rCrCrUrUrUrUrUrUrUrUrCrC/18PEG/dAdCdAdGdCdTdCdGdCdG-dGdTdTdGdAdGdGdAdCdAdA-3′ annealed to 5′-biotinyl-dTdTdGdTdCdCdTdCdAdA-dCdCdGdCdGdAdGdCdTdGdT-3' (purchased with high-performance liquid chromatography purification from Integrated DNA Technologies).

### Single-molecule FRET data acquisition and analysis

The smFRET measurements were carried out at room temperature in 50 mM HEPES, pH 7.4, 100 mM NaCl. The imaging buffer also contained an oxygen-scavenging system (0.8 mg ml^−1^ glucose oxidase, 0.625% glucose, 0.02 mg ml^−1^ catalase), 1.5 mM Trolox (used to eliminate Cy5 blinking) and 6 mM β-mercaptoethanol. The sample chamber was assembled from quartz microscope slides and glass cover slips coated with a mixture of m-PEG and biotin-PEG and pre-treated with neutravidin (0.2 mg ml^−1^). Surface tethering of doubly labelled U2AF^65^1,2L^FRET^(Cy3/Cy5) via its His-tag (Fig. 6c–f,i,j; Supplementary Fig. 7a,b) was achieved by pre-incubating the sample chamber with 50 nM biotinyl-NTA resin (Biotin-X NTA, Biotium), pre-loaded with three-fold excess NiSO_4_) for 20 min before addition of 5 nM U2AF^65^1,2L^FRET^(Cy3/Cy5). After 10 min, unbound sample was removed by washing the sample chamber with imaging buffer. The AdML RNA ligand was added to the imaging buffer at a concentration of 5 μM (100-fold higher than the measured *K*_D_ value), whereas the rArA-interrupted RNA was added at a concentration of 10 μM. Alternatively, to detect binding of doubly labelled U2AF^65^1,2L^FRET^(Cy3/Cy5) to surface-tethered RNA ligand (Fig. 6g,h Supplementary Fig. 7c–g), 10 nM AdML RNA (pre-annealed to biotinyl-DNA primer) was incubated in the neutravidin-treated sample chamber for 20 min, and 1 nM U2AF^65^1,2L^FRET^(Cy3/Cy5) was then added to the imaging buffer.

Single-molecule FRET measurements were taken as previously described^28^,^49^. An Olympus IX71 inverted microscope, equipped with a UPlanApo 60x/1.20w objective lens, a 532 nm laser (Spectra-Physics) for excitation of Cy3 dyes, and a 642 nm laser (Spectra-Physics) for excitation of Cy5 dyes was used. Total internal reflection (TIR) was obtained by a quartz prism (ESKMA Optics). Fluorescence emission was split into Cy3 and Cy5 fluorescence using a dual view imaging system DV2 (Photometrics) equipped with a 630 nm dichroic mirror and recorded *via* an Andor iXon+ EMCCD camera. Movies were recorded using the Single software (downloaded from Prof. Taekjip Ha's laboratory website at the University of Illinois at Urbana-Champaign, physics.illinois.edu/cplc/software), with the exposure time set at 100 ms. We typically took up to five 5-minute-long movies while imaging different sections of the slide for each sample. Before each measurement, we checked for non-specific binding by adding doubly-labeled U2Fret to the slide in the absence of neutravidin and imaging the slide. Non-specific binding was virtually absent.

Collected data sets were processed with IDL and Matlab softwares, using scripts downloaded from a freely available source: physics.illinois.edu/cplc/software. Apparent FRET efficiencies (*E*_app_) were calculated from the emission intensities of donor (*I*_*Cy3*_) and acceptor (*I*_*Cy5*_) as follows: *E*_app_=*I*_*Cy5*_/(*I*_*Cy5*_+*I*_*Cy3*_). The FRET distribution histograms were built from traces that showed single-step photobleaching in both Cy3 and Cy5 signals using a Matlab script generously provided by Prof. Peter Cornish (University of Missouri, Columbia). Anti-correlated changes in donor and acceptor intensities with constant sum of intensities indicated the presence of an energy transfer in single molecules labelled with one donor and one acceptor dye. All histograms were smoothed with a five-point window and plotted using Origin software (Origin Lab Co). Idealization of FRET trajectories was done using the hidden Markov model algorithms via HaMMy software (http://bio.physics.illinois.edu/HaMMy.asp)^50^. Transition density plots were generated from transitions detected in idealized FRET trajectories obtained by HaMMy fit of raw FRET traces via Matlab. Frequency of transitions from starting FRET efficiency value (*x*-axis) to ending FRET efficiency value (*y*-axis) was represented by a heat map. The range of FRET efficiencies from 0 to 1 was separated in 200 bins. The resulting heat map was normalized to the most populated bin in the plot; the lower- and upper-bound thresholds were set to 20% and 100% of the most populated bin, respectively.

The surface contour plots were generated as follows: the individual single-molecule FRET traces (for example, Fig. 6g of the main text and Supplementary Fig. 7e,f) were post synchronized at the first time point showing non-zero (>0.15) FRET efficiency, corresponding to binding. The time range (*x*-axis, 0–10 s) was separated into 100 bins. The FRET efficiency range (*y*-axis, 0–1 FRET) was separated into 100 bins. A heat map is used to represent the frequency of sampling of each FRET state over time; frequency in each bin was normalized to the most populated bin in the plot with lower- and upper-bound thresholds set at 10% and 80% of the most populated bin, respectively.

## Additional information

**Accession codes:** Coordinates and structure factors have been deposited in the Protein Data Bank with accession codes 5EV1, 5EV2, 5EV3 and 5EV4 for respective U2AF^65^1,2L-oligonucleotide structures (i)–(iv).

**How to cite this article:** Agrawal, A. A. *et al.* An extended U2AF^65^–RNA-binding domain recognizes the 3′ splice site signal. *Nat. Commun.* 7:10950 doi: 10.1038/ncomms10950 (2016).

## Supplementary Material

Supplementary Information Supplementary Figures 1-8, Supplementary Discussion and Supplementary References.

Supplementary Movie 1 Overall structure and central nucleotide interactions.

## Figures and Tables

**Table 1:** Crystallographic data and refinement statistics*.

**Structure**	**U2AF**^**65**^**1,2L with rUrUrUdUdU(BrdU)dUrUrU**	**U2AF**^**65**^**1,2L with (P)rUrUdUdUrUdU(BrdU)dU**	**U2AF**^**65**^**1,2L with (P)rUrUdUrUrU(BrdU)dUdU**	**U2AF**^**65**^**1,2L with (P)rUrUrUdUrUrU(BrdU)dUrC**
*Data collection*	(i)	(ii)	(iii)	(iv)
Space group	C222_1_	C222_1_	P2_1_2_1_2_1_	P2_1_2_1_2_1_
Unit cell (Å) *a,b,c*	62.1, 114.2, 59.4	61.9, 115.1, 59.5	43.4, 62.2, 77.4	43.5, 63.4, 77.7
Resolution limits (Å)	32.46–2.04	32.57–1.86	38.71–1.50	38.83–1.57
Completeness (%)	95.5 (78.3)	98.7 (95.9)	98.2 (69.8)	98.3 (71.7)
Redundancy	4.6 (4.1)	4.3 (4.2)	6.1 (3.0)	6.2 (3.1)
*I*/*σ*(*I*)	21.2 (4.2)	24.6 (4.6)	38.0 (6.5)	42.9 (6.9)
*R*_sym_ (%)	3.9 (32.1)	3.9 (30.3)	2.4 (14.8)	2.2 (14.9)
*Refinement*				
No. reflections (work/test)	12,124/1,055	17,870/1,456	31,802/1,996	28,162/2,000
*R*_work_/*R*_free_ (%)	17.3/22.8	15.1/18.8	15.3/18.6	15.4/17.6
*No. atoms*				
Protein	2,982	3,052	2,986	2,978
Oligonucleotide	214	209	198	255
Water	118	203	263	177
*Bond r.m.s.d.*				
Bond lengths (Å)	0.013	0.010	0.008	0.009
Bond angles (°)	1.32	1.1	1.05	1.05
*<B> factors (Å^2^)*				
Protein	46.4	27.4	26.3	26.7
Oligonucleotide	61.8	35.2	24.5	30.5
Water	45.2	35.2	30.7	29.8

All available crystallographic data was used for refinement.

^*^A single crystal was used for each structure. Values from the highest resolution shell are given in parentheses: 2.15–2.04; 1.90–1.86; 1.53–1.50; 1.61–1.57.
