# Supplementary material for: An extended U2AF65–RNA-binding domain recognizes the 3′ splice site signal
Source: Nat Commun. 2016 Mar 8;7:10950. doi: 10.1038/ncomms10950 (PMC4786784; doi:10.1038/ncomms10950)
Supplement: Supplementary Information — Supplementary Figures 1-8, Supplementary Discussion and Supplementary References. [file ncomms10950-s1.pdf]

Supplementary Figures and Legends

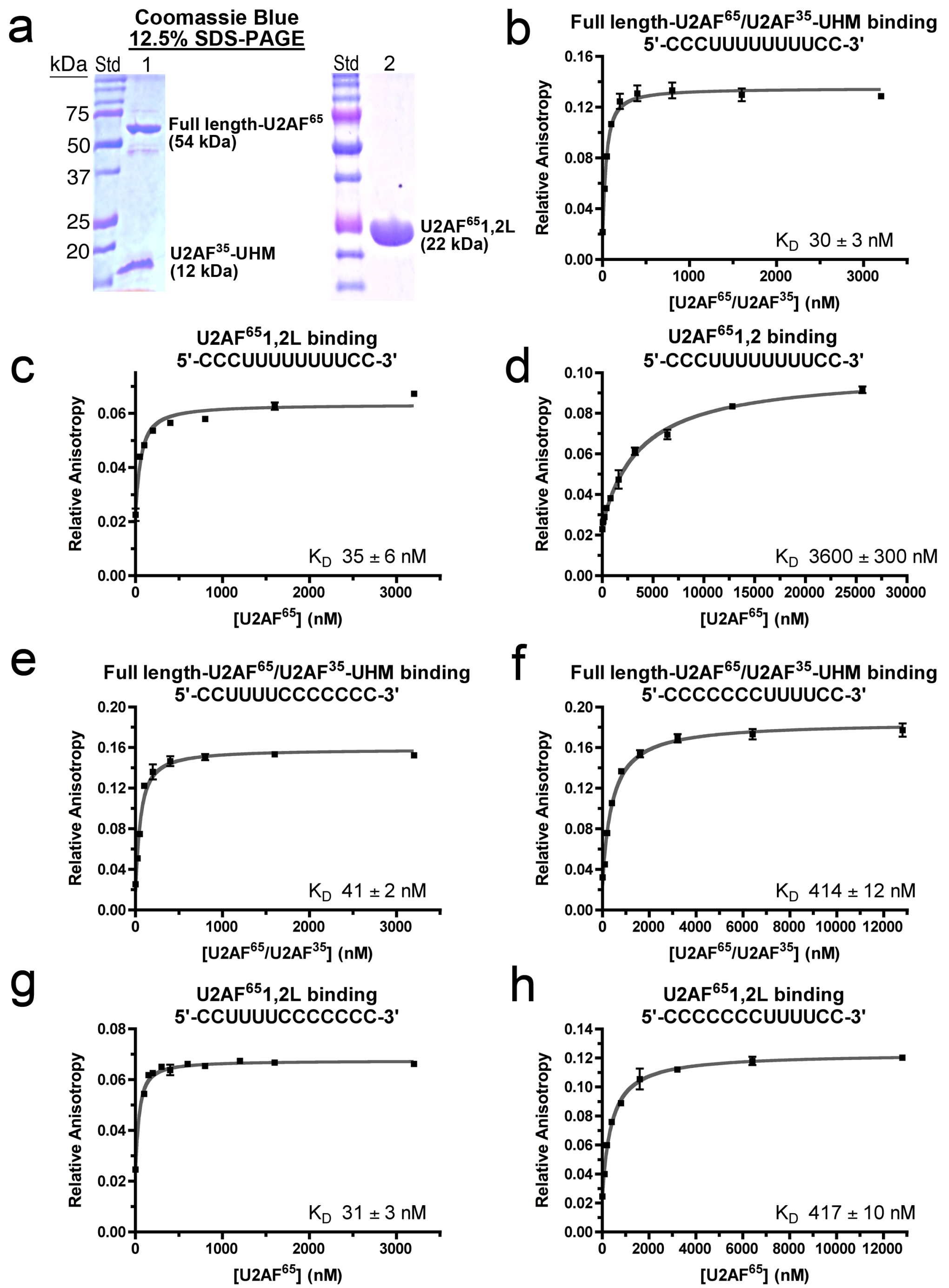

Supplementary Figure 1. Fluorescence anisotropy RNA binding curves demonstrating that residues flanking the core U2AF[65] RRMs are necessary to re-capitulate Py tract recognition compared with the full length U2AF[65]. (**a**) Purified proteins analyzed by 12.5% SDS-PAGE followed by Coomassie-blue staining. Molecular weight standards marked to the left are the same for both gels. (**b-h**) The average data points and error bars of three independent fluorescence anisotropy binding experiments are overlaid with the nonlinear fits as described[1]. The protein constructs and RNA sequences tested are indicated in the graph title. The apparent equilibrium dissociation constants (K_D) are inset.

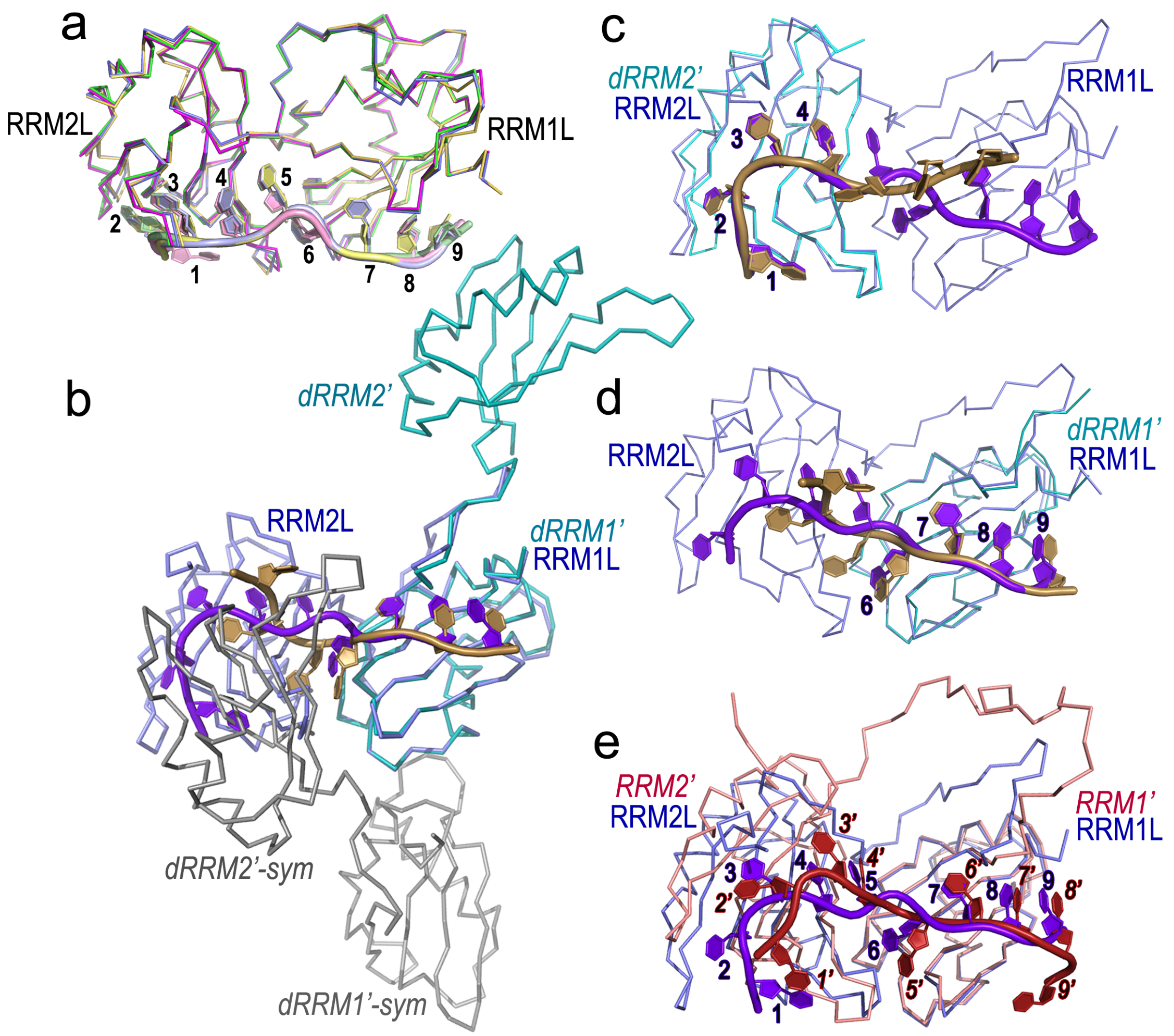

Supplementary Figure 2. Comparison among U2AF^{65} structures. (**a**) Superposition of U2AF^{65}1,2L structures *(i)* – *(iv)* by matching Cα atoms. (**b**) Overlay of U2AF^{65}1,2L bound to 5´-(P)rUrUrUdUrUrU(BrdU)dUrC (slate) and dU2AF^{65}1,2 (cyan, labeled in primed italics) bound to rU 7-mer (gold) superposed by match Cα atoms of RRM1. The symmetry-related dU2AF^{65}1,2 polypeptide that binds with the 5´ region of the rU 7-mer is shown (gray, *sym*). The oligonucleotide bound to RRM1 of the symmetry-related dU2AF^{65}1,2 polypeptide is omitted for clarity. (**c**) Overlay of U2AF^{65}1,2L bound to 5´-(P)rUrUrUdUrUrU(BrdU)dUrC (slate) and RRM2 of dU2AF^{65}1,2 (cyan, *dRRM2'*) bound to rU 7-mer (gold). (**d**) Overlay of U2AF^{65}1,2L bound to 5´-rUrUrUdUdU(5BrdU)dUrUrU (slate) and RRM1 of dU2AF^{65}1,2 (cyan, *dRRM1'*) bound to rU 7-mer (gold). Matching bound nucleotides are numbered in b and c. (**e**) Overlay of U2AF^{65}1,2L bound to 5´-(P)rUrUrUdUrUrU(BrdU)dUrC (slate) and NMR structure of U2AF^{65}1,2 bound to rU 9-mer (PDB ID 2YH1, red) superposed by matching Cα atoms in RRM1. Bound nucleotides are numbered and distinguished by primed italics for the NMR structure.

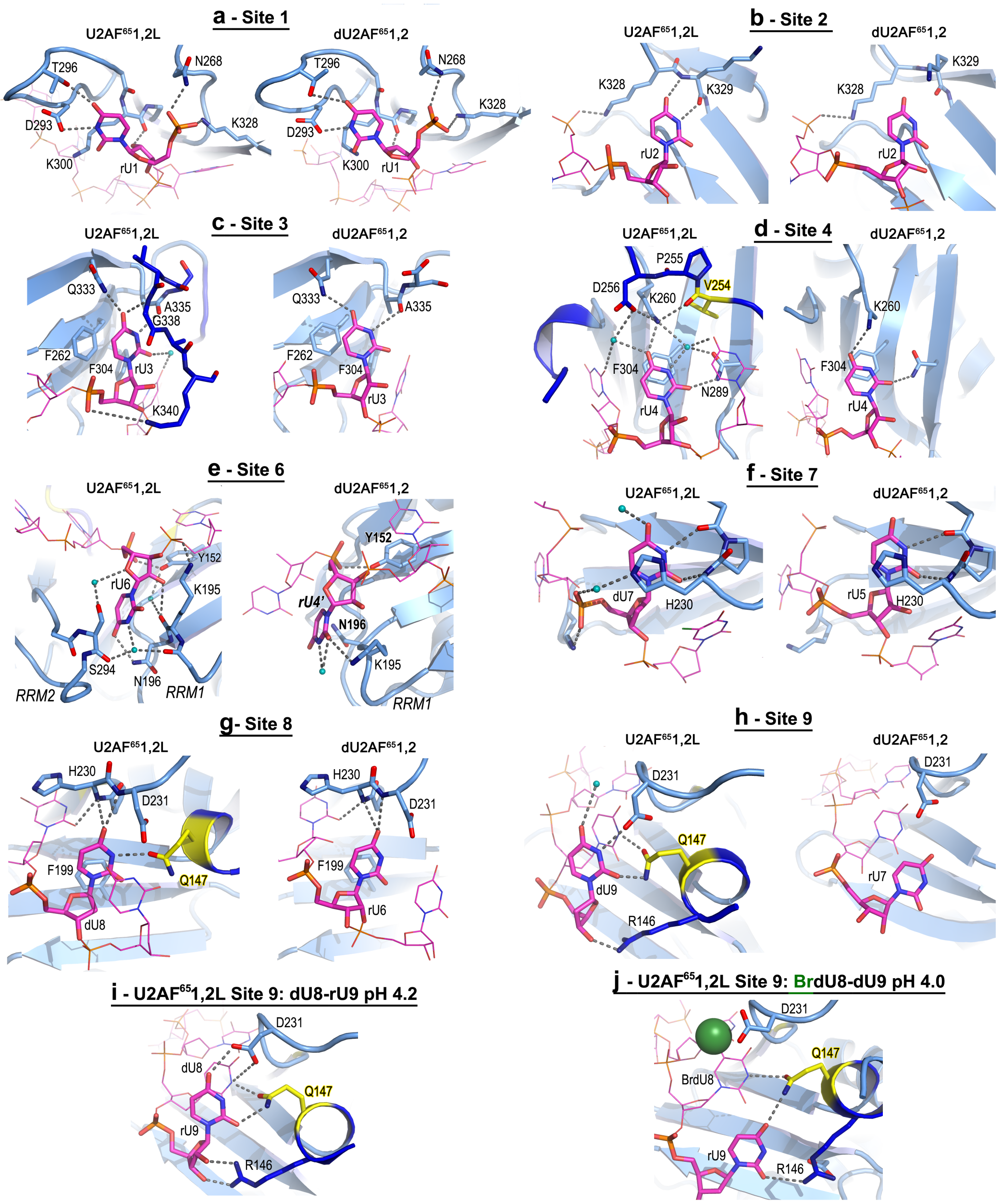

Supplementary Figure 3. Comparison of the Py tract binding sites of U2AF^{65}1,2L at neutral pH with (**a-h**) dU2AF^{65}1,2 at neutral pH (PDB ID 2G4B)and (**i-j**) U2AF^{65}1,2L at low pH. Hydrogen bonding atoms within <3.5 Å are connected by dashed lines. Nucleotides are numbered to match the corresponding PDB; U2AF^{65}1,2L is bound to nine nucleotides whereas dU2AF^{65}1,2 is bound to seven. With the exception of equivalent interactions at the first and seventh binding sites, which shown for (**a**) rU1 of U2AF^{65}1,2L structure *i* and (**f**) dU7 of U2AF^{65}1,2L structure *ii*,
new nucleotides interactions are evident at the majority of U2AF^{65}1,2L sites and are represented as for Fig. 3. The dU2AF^{65}1,2 lacks the fifth binding site. The so-called dU2AF^{65}1,2 fourth (**d**) and sixth (**e**) binding sites are related by crystal packing and sandwich the same nucleotide (rU4 and *rU4'*), yet generally match the locations of the respective U2AF^{65}1,2L binding sites. (**i**) Protonation of the D231 carboxylate at the low pH of U2AF^{65}1,2L structure *i* promotes hydrogen bond formation with the terminal rU9. (**j**) Intrusion of the bulky bromine (green sphere) in 5Br-dU8 unstacks the terminal rU9 of U2AF^{65}1,2L structure *ii*.

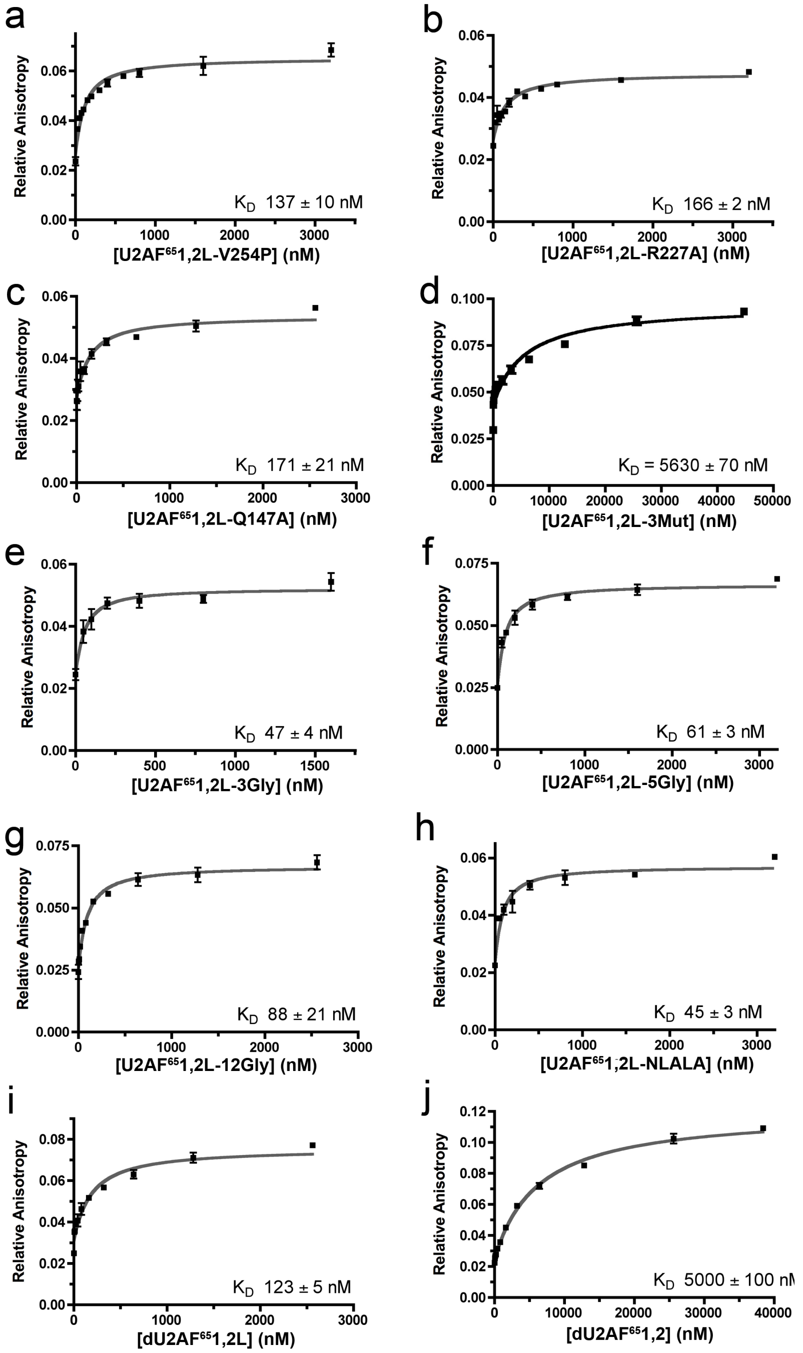

Supplementary Figure 4. Fluorescence anisotropy RNA binding curves that test the contribution of U2AF^{65} linker residues to recognition of a representative Py tract. The average data points and error bars of three independent experiments are overlaid with the nonlinear fits as described[1]. Proteins are titrated into 5′-fluorescein-labeled AdML Py tract RNA (5′-CCCUUU UUUUUCC). The U2AF^{65}12L protein variants contain the following mutations: (**a**) V254P, (**b**) R227A, (**c**) Q147A, (**d**) V254P/R227A/Q147A (3Mut), (**e**) V249G/V250G/V254G (3Gly), (**f**) S251G/T252G/V253G/V254G/P255G (5Gly), (**g**) M144G/L235G/M238G/V244G/V246G/V249G/V250G/S251G/T252G/V253G/V254G/P255G (12Gly), (**h**) S251N/T252L/V253A/V254L/P255A (NLALA), or the internal deletion of residues 238-257 in the context of either (**j**) the U2AF^{65}12L boundaries (dU2AF^{65}12L) or (**i**) the minimal RRM1-RRM2 core (dU2AF^{65}12).

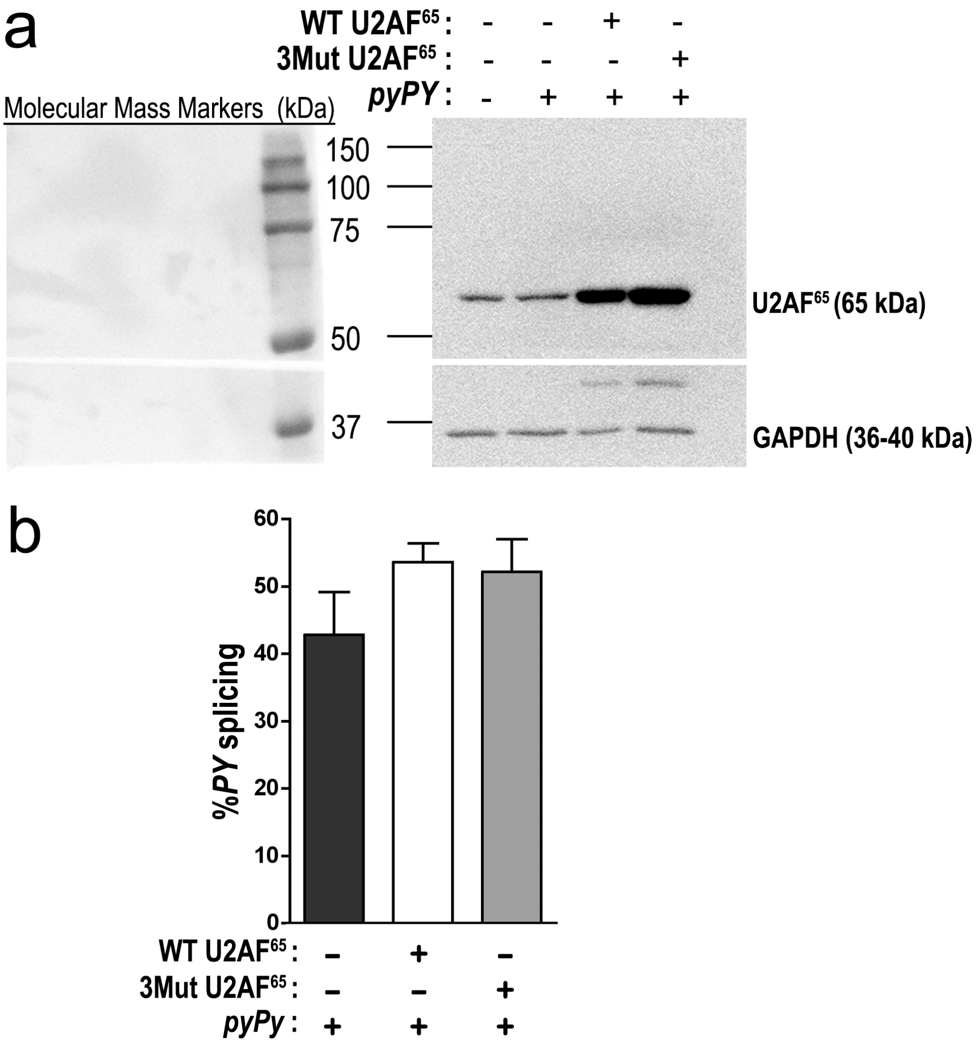

Supplementary Figure 5. Supplementary data for new U2AF^{65} interactions are important for *pyPY* splicing in human cells. (**a**) Immunoblot (right) showing similar levels of wild-type (WT) U2AF^{65} and triple Q147A/R227A/V254P mutant (3Mut) U2AF^{65} expression following transient co-transfection of HEK 293T cells with or without the *pyPY* minigene. White lines mark where the PVDF membrane was cut for immunoblotting with different antibodies, which include mouse monoclonal antibodies directed against U2AF^{65} (MC3, Cat. No. U4758 Sigma-Aldrich at 1:500 dilution) or as a loading control, against GAPDH (monoclonal clone 71.1, Cat. No. G8795 Sigma-Aldrich at 1:10,00 dilution). A fluorescent scan of the identical immunoblot membrane shows the positions of molecular weight standards (left). (**b**) A bar graph of the average percentage and standard deviations among four independent biological replicates of the *PY*-spliced mRNA relative to total *pyPY* transcripts (spliced and unspliced) detected by RT-PCR (black, no U2AF^{65} added; white, WT U2AF^{65}; gray, 3Mut U2AF^{65}).

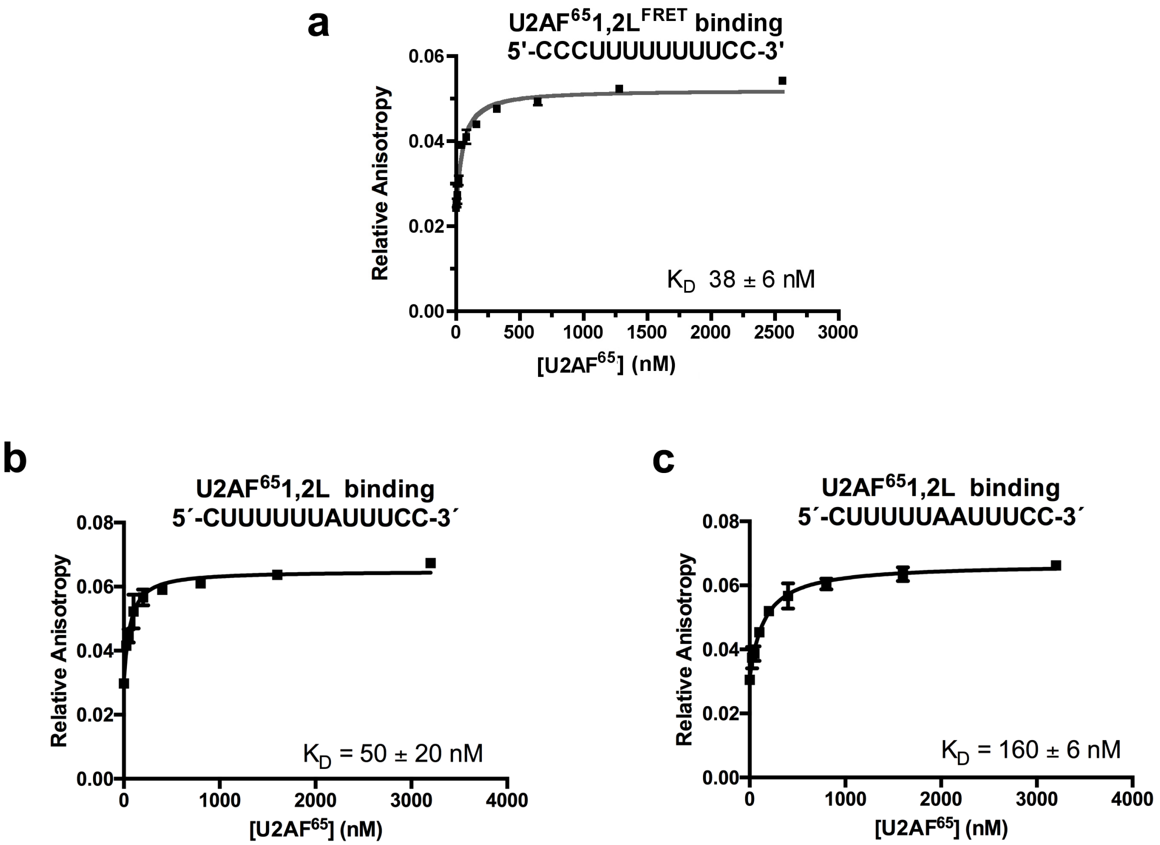

Supplementary Figure 6. (**a**) Fluorescence anisotropy RNA binding curve for U2AF^{65}1,2L^{FRET} protein binding 5´-fluorescein-labeled AdML Py tract RNA. (**b-c**) Fluorescence anisotropy RNA binding curves for U2AF^{65}1,2L protein binding 5´-fluorescein-labeled A-interrupted RNAs (sequence inset). The average data points and error bars of three independent experiments are overlaid with the nonlinear least square fits and the apparent equilibrium dissociation constants (K_D) are inset.

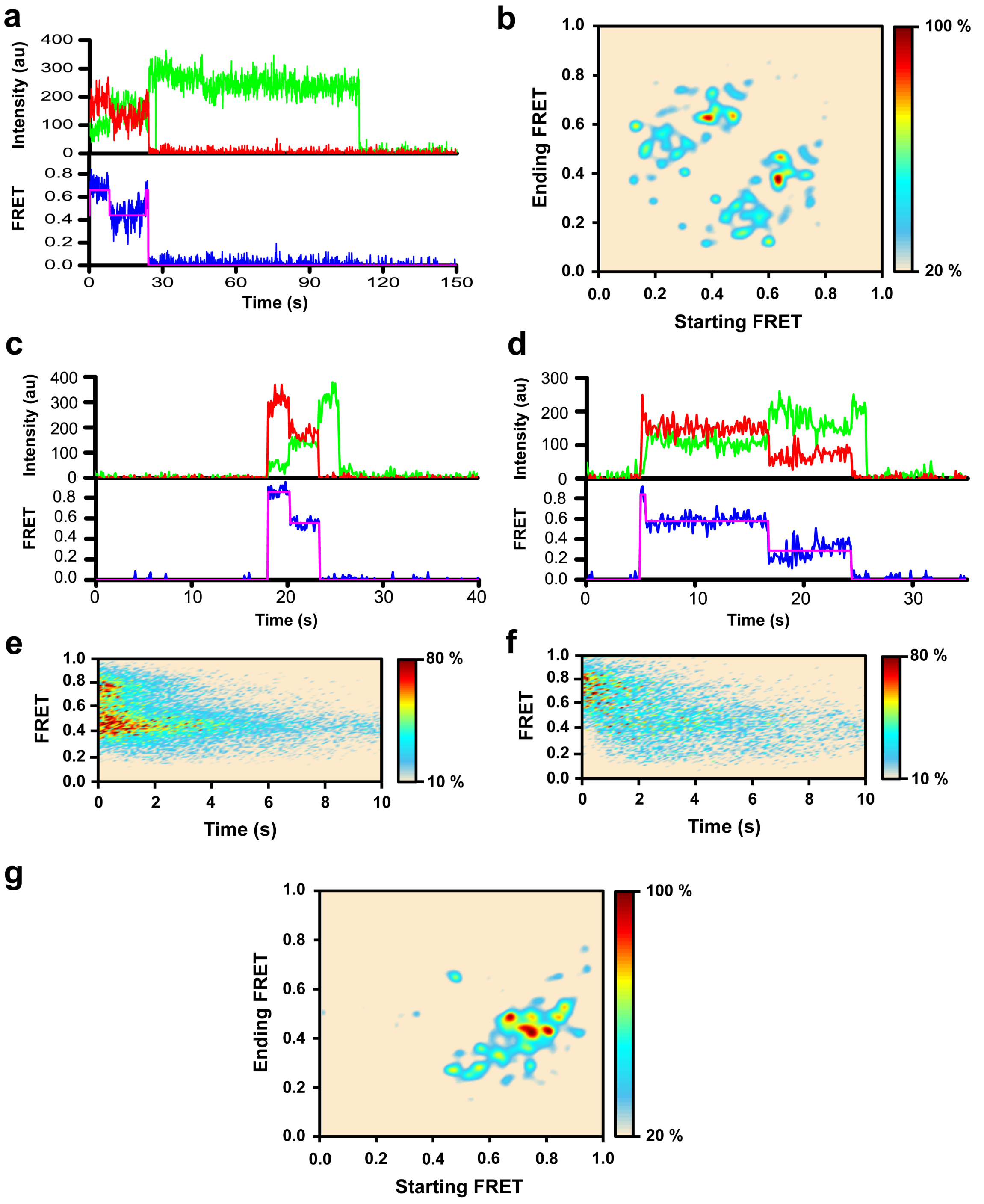

Supplementary Figure 7. Analysis of fluctuations of U2AF^{65}1,2L^{FRET} between different FRET values. **(a-b)** The U2AF^{65}1,2L^{FRET}(Cy3/Cy5) protein was immobilized on the microscope slide *via* biotin-NTA/Ni^{+2} resin and imaged in the absence of RNA. **(c-g)** Alternatively, U2AF^{65}1,2L^{FRET}(Cy3/Cy5) protein (1 nM) was added biotinyl AdML Py tract RNA (10 nM), which was immobilized on the microscope slide, and then imaged.

(Legend continued next page)

(Supplementary Figure 7 legend, continued)

In the representative FRET traces shown in (**a, c-d**), the fluorescence intensities of donor and acceptor fluorophores are shown in green and red, respectively; observed trajectory for FRET efficiency is shown in blue; FRET efficiency idealized by hidden Markov model analysis is shown in magenta. Single molecule FRET traces were idealized using the hidden Markov model algorithms of HaMMy software[2]. **(b, g)** Heat maps of transition density plots (TDPs) represent the frequency of transitions from the starting FRET efficiency value (x-axis) to the ending FRET efficiency value (y-axis). The range of FRET efficiencies from 0 to 1 was separated in 200 bins. The resulting heat map was normalized to the most populated bin in the plot; the lower- and upper-bound thresholds were set to 20% and 100% of the most populated bin, respectively. **(e-f)** Surface contour plots generated by superimposition of FRET traces post-synchronized at the time of $U2AF^{65}1,2L^{FRET}$(Cy3/Cy5) binding to surface-tethered biotinyl AdML RNA show the evolution of FRET over time. (**e**) A contour plot generated by superimposition of all 1050 binding events in the dataset (matching the histogram in Fig. 6h of the main text). (**f**) A contour plot generated by superimposition of only those smFRET traces (294 out of 1050) that show apparent transitions between different FRET values (these traces were also used to generate the TDP **(g)**.

(**a-b**) Although fluctuations in smFRET traces of RNA-free $U2AF^{65}1,2L^{FRET}$(Cy3/Cy5) were too infrequent to unambiguously define the number of FRET states using HaMMy analysis, the majority (80%) of traces showing fluctuations were well fit by a two-state model **(a)**. The TDP **(b)** generated from 3,500 transitions detected in 373 idealized FRET trajectories obtained by two-state HaMMy fit of raw FRET traces suggests that RNA-free $U2AF^{65}1,2L^{FRET}$(Cy3/Cy5) most frequently fluctuates between 0.6 and 0.4 FRET values.

(c-g) 294 smFRET traces for RNA-bound $U2AF^{65}1,2L^{FRET}$(Cy3/Cy5), which showed apparent fluctuations, (30% of the total number of traces, **Fig. 6g-h**) were fit to three-state model using HaMMy[2]. TDP generated from 343 detected transitions **(g)** as well as contour plots showing evolution of FRET as a function of time **(e-f)** revealed a predominantly-irreversible transition from ~0.7-0.8 to ~0.45 FRET value. Less frequently, transitions between ~0.45 and ~0.3 FRET values are also observed.

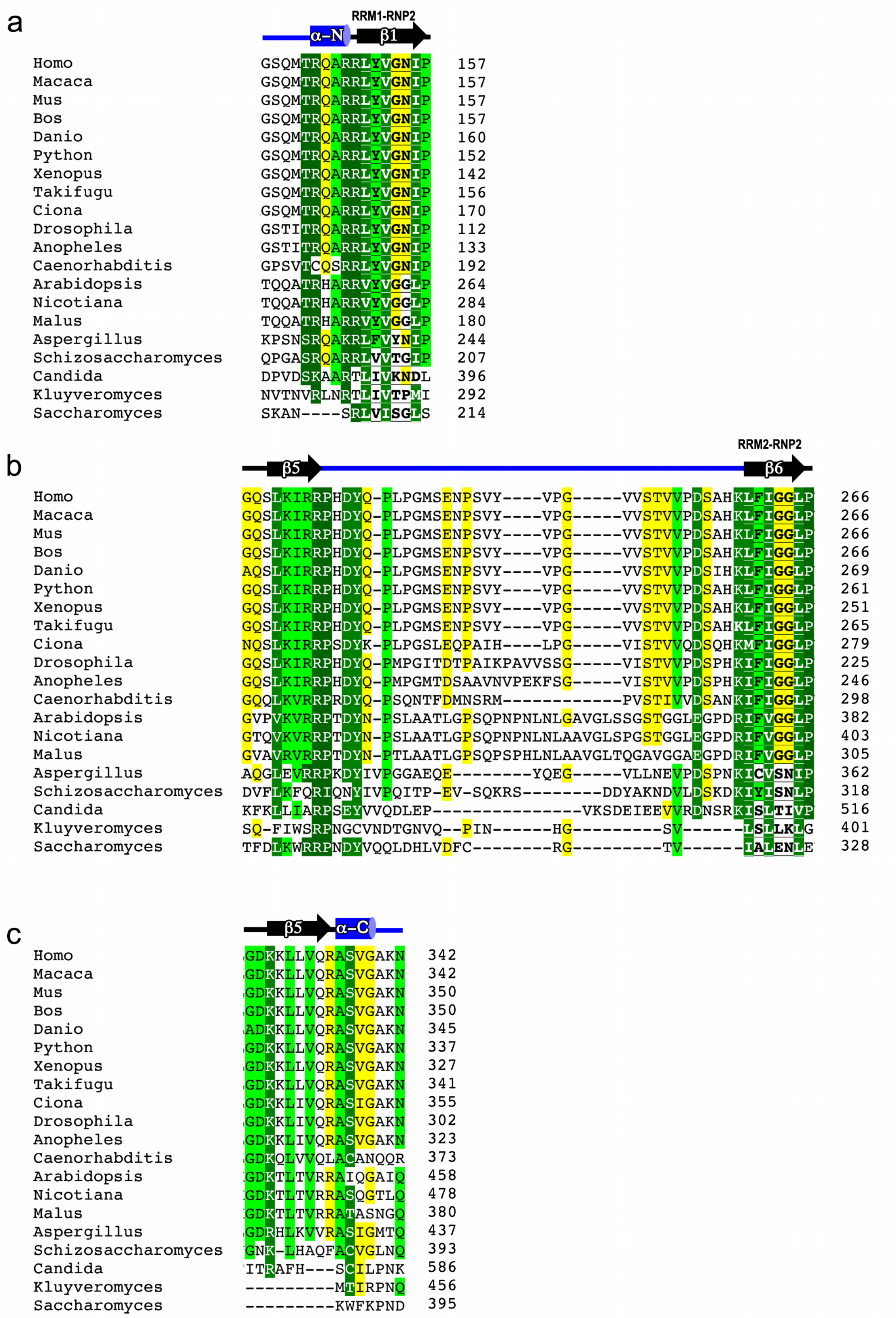

Supplementary Figure 8.

(Legend continued next page)

(Supplementary Figure 8 legend, continued)

Sequence conservation of U2AF^{65} inter-RRM regions: (**a**) N-terminal RRM1 extension, (**b**) inter-RRM linker, (**c**) C-terminal RRM2 extension. The sequences of twenty known and probable U2AF^{65} orthologues were aligned using Clustal Omega[3] and by visual inspection using secondary structure prediction. Human U2AF^{65} secondary structure elements are indicated above the aligned sequences, which are colored by sequence identity as follows: >90% dark green, >80% green, >70% yellow. The secondary structure of the U2AF^{65}1,2L structure is indicated above: rectangle, α-helix; line, coil; arrow, β-strand and colored blue for new regions (this work) and black for secondary structures assigned in reference[4]. The new N- and C-terminal α-helices are labeled α-N and α-C, respectively. Ribonucleoprotein consensus motifs (RNP1 and RNP2) are labeled. Aligned orthologues include: *Homo sapiens* (NCBI Refseq NP_001012496), *Macaca mulatta* (NCBI Refseq XP_001119590), *Mus musculus* (NCBI Refseq NP_001192160), *Bos Taurus* (NCBI Refseq NP_001068804), *Python bivittatus* (NCBI Refseq XP_007432990), *Xenopus laevis* (NCBI Refseq NP_001080595), *Danio rerio* (NCBI Refseq XP_009292312), *Takifugu rubripes* (NCBI Refseq XP_011604148), *Ciona intestinalis* (NCBI Refseq XP_002130386), *Drosophila melanogaster* (NCBI Refseq NP_001245708), *Anopheles gambiae* (NCBI Refseq XP_311994), *Caenorhabditis elegans* (NCBI Refseq NP_001022967), *Arabidopsis thaliana* (NCBI Refseq NP_176287), *Nicotiana sylvestris* (NCBI Refseq XP_009804080), *Malus domestica* (NCBI Refseq XP_008383769), *Aspergillus niger* (NCBI Refseq XP_001391373), *Schizosaccharomyces pombe* (NCBI Refseq NP_595396), *Candida albicans* (NCBI Refseq XP_715304), *Kluyveromyces lactis* (NCBI Refseq XP_456258), *Saccharomyces cerevisiae* (NCBI Refseq NP_012849).

Supplementary Discussion

Comparison of U2AF^{65}1,2L and d U2AF^{65}1,2 (PDB ID 2G4B). The arrangement of the RNA-bound U2AF^{65}1,2L RRM1 and RRM2 differs completely from the dU2AF^{65}1,2 structure[4] (RMSD 17.2 Å for 156 matching Cα atoms), for which each dU2AF^{65}1,2 RRM of a given polypeptide interacts with a separate oligonucleotide (**Supplementary Fig. 2b**). Nevertheless, the folds of the individual U2AF^{65}1,2L and dU2AF^{65}1,2 RRMs and a subset of the nucleotide interactions are similar (RMSD 0.5 Å/1.0 Å for 77/79 respective RRM1/RRM2 Cα atoms) (**Supplementary Fig. 2c-d**).

Comparison of U2AF^{65}1,2L and U2AF^{65}1,2 (PDB ID 2YH1). The relative positions of the core RRM1/RRM2 in our U2AF^{65}1,2L crystal structures agree with the PRE/NMR-based, RNA-bound U2AF^{65}1,2 structure (RMSD 2.9 Å for 156 RRM1/RRM2 Cα atoms) (**Supplementary Fig. 2e**). The inter-RRM linkers of the prior U2AF^{65}1,2 structures are *ab initio* models for which experimental restraints were omitted[5]. Moreover, the unique N- and C-terminal extensions of the U2AF^{65}1,2L structures appear to stabilize the structure of the inter-RRM linker. Accordingly, the models of the U2AF^{65}1,2 inter-RRM linker differ from the current U2AF^{65}1,2L structures (RMSD 8.5 Å for 193 matching Cα atoms of the RNA-bound U2AF^{65}1,2 NMR structure, PDB ID 2YH1 and U2AF^{65}1,2L structure *iv*). The bound Py tract of the U2AF^{65}1,2 NMR structure also digresses from the U2AF^{65}1,2L structure in conformation and placement of the nucleotide binding sites, most likely due to the reliance of the NMR structure on the discontinuous dU2AF^{65}1,2 RRM structures for restraints.

Comparison of U2AF^{65} bound to uracil *versus* cytosine at the ninth binding site. Comparison among the structures shows how U2AF^{65} adapts to uracil compared with cytosine pyrimidines at the ninth binding site (**Fig. 3g-h**). When interacting with uracil in structure *iii*, the D231 carboxylate accepts a hydrogen bond from the dU9-N3H. Following substitution to cytosine in structure *iv*, the

D231 side chain rotates to accept a hydrogen bond with the rC9-N4H_2 exocyclic amine. The U2AF^{65}12L structures *iii* and *iv* bound to dU9 and rC9 were determined at near physiological pH (pH 7.0). At low pH (pH 4.2 in structure *i*), the protonated D231 side chain shifts to donate a hydrogen bond to the rU9-O4 as well as the dU8-N3H of the preceding nucleotide (**Supplementary Fig. 3i**). Addition of the bulky bromine to the preceding BrdU8 base unstacks and relocates the dU9 uracil (in structure *ii*, **Supplementary Fig. 3j**), which could explain the preference of U2AF^{65} to bind BrdU at the seventh site (**Fig. 2a** and reference[6]). Otherwise, the sugar moiety shares similar positions among the rC9, rU9, and dU9 nucleotides preceded by dU8, indicating that the dU9 of structure *iii* is likely to accurately reflect the conformation for rU9 at neutral pH. The unfavorable proximity of the D231 side chain to the rU8-O4 is consistent with the preference of a valine substitution (U2AF^{65}-D231V) to bind uridines at the eighth site[7]. Likewise, hydrogen bonds between the U2AF^{65}-D231 side chain and the exocyclic amines of terminal cytosines could underlie UUUUUUUCC enrichment following *in vitro* selection experiments with U2AF^{65} [8].

Function and conservation of the U2AF^{65} inter-RRM linker. We show through a series of high resolution structures that U2AF^{65} recognizes a contiguous nine-nucleotide Py tract. The structures and structure-guided biochemistry reveal that residues surrounding the core RRM1 and RRM2 are integral for Py tract recognition. Well-ordered α-helices extend the N- and C-termini of RRM1 and RRM2 and directly recognize the respective ninth and third nucleotides of the Py tract. The inter-RRM linker comprises a central nucleotide-binding site and contributes to the preceding, fourth nucleotide-binding site.

A primary constraint for the RNA binding functions of the U2AF^{65} inter-RRM linker appears to be the protein backbone capacity to form hydrogen bonds with the bound nucleobase. A single mutation (V254P) abolishes hydrogen bond formation between the linker backbone atoms and the

central nucleotide and significantly reduces U2AF^{65} – RNA affinity. Little consequence for RNA binding is observed following up to 12 substitutions of U2AF^{65} linker residues with glycine, which is capable of hydrogen bond formation with the central nucleotide.

The sequence-insensitive intra-molecular contacts of the U2AF^{65} inter-RRM linker agree with its low primary sequence conservation (**Supplementary Fig. 8**). Within the central portion of the linker, the V254 residue that directly recognizes the Py tract is the sole linker residue that is nearly identical among documented U2AF^{65} homologues. The D256 residue that indirectly contributes to the fourth binding site is a second rare example of a highly conserved U2AF^{65} inter-RRM linker residue, for which a similar change to E is the only common variation among homologues from multicellular organisms. A few conserved residues that serve structural roles in the linker conformation include R228, P229, Y232, and P234 at the initial turn abutting RRM1, and G248 at the apex of the linker between RRM1 and RRM2. Otherwise, the inter-RRM linker sequence composition is variable among U2AF^{65} homologues (**Supplementary Fig. 8b**). The RNA-interacting residues of the U2AF^{65} RRM extensions also are highly conserved (**Supplementary Fig. 8a,c**). For example, the R146 residue that recognizes the terminal pyrimidine base is strictly conserved among U2AF^{65} homologues from multicellular organisms with the exception of *Caenorhabditis elegans*, which may be related to the unusually short, consensus Py tract of this organism. Likewise, U2AF^{65}-Q147 is either conserved or replaced by a histidine residue that is capable of similar hydrogen bond interactions. These selective trends in sequence conservation support the importance of these U2AF^{65} contacts and conformation for recognition of the central Py tract nucleotides.

Within limits, the length of the inter-RRM linker also varies among U2AF^{65} homologues (**Supplementary Fig. 8b**). The length of the human U2AF^{65} linker lies at a midpoint of approximately 30 residues. In comparison, the inter-RRM linkers of *Kluyveromyces lactis* and *Saccharomyces cerevisiae* U2AF^{65} homologues are unusually short (~17 residues). This suggests that these yeast

U2AF[65] homologues may adopt a primordial mode of Py tract recognition. Accordingly, *K. lactis* and *S. cerevisiae* lack alternative splicing and are set apart from other fungi by a clear U-enrichment near the 3´ end of introns[9]. Conversely, the inter-RRM linkers of U2AF[65] homologues from multicellular plants are unusually long (e.g. 40 residues in *Arabidopsis thaliana* U2AF[65]), show increased contents of the serine handle for post-translational modification, and typically replace the V254 counterpart with glycine. These differences suggest divergent and potentially regulated modes of U2AF[65] – Py tract recognition in plants, for which alternative splicing serves key functions in multicellular plant development, defense, and stress response[10,11].

Supplementary References:

1. Jenkins, J.L., Shen, H., Green, M.R. & Kielkopf, C.L. Solution conformation and thermodynamic characteristics of RNA binding by the splicing factor U2AF^{65}. *J Biol Chem* **283**, 33641-33649 (2008).
2. McKinney, S.A., Joo, C. & Ha, T. Analysis of single-molecule FRET trajectories using hidden Markov modeling. *Biophys J* **91**, 1941-51 (2006).
3. Sievers, F. et al. Fast, scalable generation of high-quality protein multiple sequence alignments using Clustal Omega. *Mol Syst Biol* **7**, 539 (2011).
4. Sickmier, E.A. et al. Structural basis of polypyrimidine tract recognition by the essential pre-mRNA splicing factor, U2AF^{65}. *Mol Cell* **23**, 49-59 (2006).
5. Mackereth, C.D. et al. Multi-domain conformational selection underlies pre-mRNA splicing regulation by U2AF. *Nature* **475**, 408-11 (2011).
6. Jenkins, J.L., Agrawal, A.A., Gupta, A., Green, M.R. & Kielkopf, C.L. U2AF^{65} adapts to diverse pre-mRNA splice sites through conformational selection of specific and promiscuous RNA recognition motifs. *Nucleic Acids Res* **41**, 3859-73 (2013).
7. Agrawal, A.A., McLaughlin, K.J., Jenkins, J.L. & Kielkopf, C.L. Structure-guided U2AF^{65} variant improves recognition and splicing of a defective pre-mRNA. *Proc Natl Acad Sci U S A* **111**, 17420-5 (2014).
8. Singh, R., Valcarcel, J. & Green, M.R. Distinct binding specificities and functions of higher eukaryotic polypyrimidine tract-binding proteins. *Science* **268**, 1173-1176. (1995).
9. Schwartz, S.H. et al. Large-scale comparative analysis of splicing signals and their corresponding splicing factors in eukaryotes. *Genome Res* **18**, 88-103 (2008).
10. Reddy, A.S., Marquez, Y., Kalyna, M. & Barta, A. Complexity of the alternative splicing landscape in plants. *Plant Cell* **25**, 3657-83 (2013).
11. Staiger, D. & Brown, J.W. Alternative splicing at the intersection of biological timing, development, and stress responses. *Plant Cell* **25**, 3640-56 (2013).
